# RFARN: Retinal vessel segmentation based on reverse fusion attention residual network

**DOI:** 10.1371/journal.pone.0257256

**Published:** 2021-12-03

**Authors:** Wenhuan Liu, Yun Jiang, Jingyao Zhang, Zeqi Ma

**Affiliations:** College of Computer Science and Engineering, Northwest Normal University, Lanzhou Gansu, China; University of Engineering & Technology, Taxila, PAKISTAN

## Abstract

Accurate segmentation of retinal vessels is critical to the mechanism, diagnosis, and treatment of many ocular pathologies. Due to the poor contrast and inhomogeneous background of fundus imaging and the complex structure of retinal fundus images, this makes accurate segmentation of blood vessels from retinal images still challenging. In this paper, we propose an effective framework for retinal vascular segmentation, which is innovative mainly in the retinal image pre-processing stage and segmentation stage. First, we perform image enhancement on three publicly available fundus datasets based on the multiscale retinex with color restoration (MSRCR) method, which effectively suppresses noise and highlights the vessel structure creating a good basis for the segmentation phase. The processed fundus images are then fed into an effective Reverse Fusion Attention Residual Network (RFARN) for training to achieve more accurate retinal vessel segmentation. In the RFARN, we use Reverse Channel Attention Module (RCAM) and Reverse Spatial Attention Module (RSAM) to highlight the shallow details of the channel and spatial dimensions. And RCAM and RSAM are used to achieve effective fusion of deep local features with shallow global features to ensure the continuity and integrity of the segmented vessels. In the experimental results for the DRIVE, STARE and CHASE datasets, the evaluation metrics were 0.9712, 0.9822 and 0.9780 for accuracy (Acc), 0.8788, 0.8874 and 0.8352 for sensitivity (Se), 0.9803, 0.9891 and 0.9890 for specificity (Sp), area under the ROC curve(AUC) was 0.9910, 0.9952 and 0.9904, and the F1-Score was 0.8453, 0.8707 and 0.8185. In comparison with existing retinal image segmentation methods, e.g. UNet, R2UNet, DUNet, HAnet, Sine-Net, FANet, etc., our method in three fundus datasets achieved better vessel segmentation performance and results.

## Introduction

**Fig 1:**
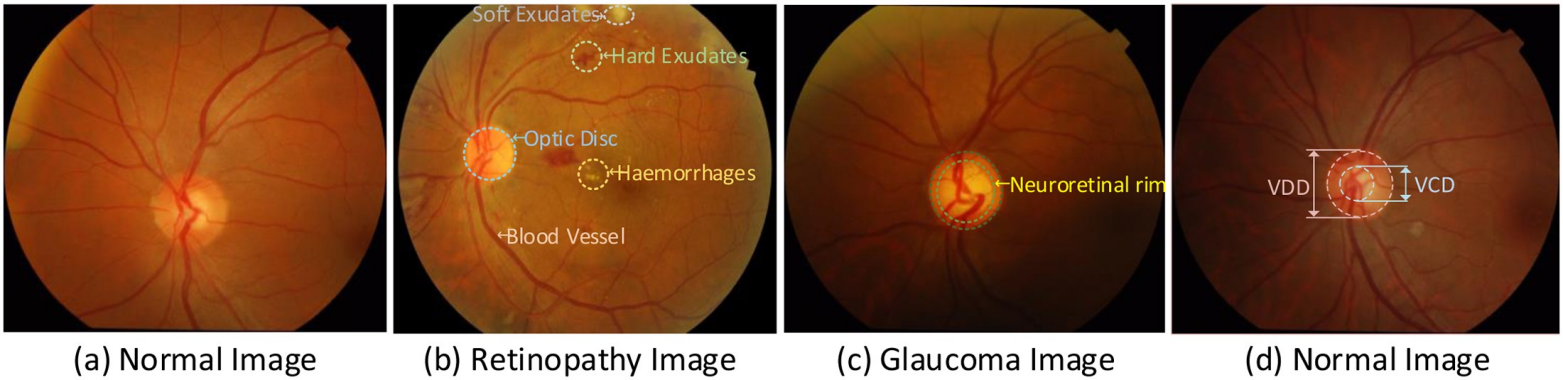
Fundus retinal image structure. (a) is a healthy fundus image; (b) is an image of diabetic retinopathy with a large amount of hemorrhage and exudate; (c) is an image of glaucoma with a narrower neuroretinal rim band between the optic disc and the optic cup; (d) is also a normal fundus image with a small VCD to VDD ratio.

Image segmentation is one of the most studied problems in computer vision, where the main goal is to classify each pixel of an image into a specific class of instances [1]. In the field of medical ophthalmology, the goal of segmentation is to accurately classify the blood vessels and background pixels of a patient’s fundus image. Glaucoma, diabetic retinopathy, age-related macular degeneration and fundus retinopathy are all diseases in the field of ophthalmology. In these, diabetic retinopathy is a major cause of blindnes [2]. The retinal vasculature is again the only deep microvasculature in the blood circulation system which can be directly and non-invasively visualized. It is extremely rich in information about its vascular characteristics [3]. More importantly, the morphological information related to the retinal vascular tree (e.g. curvature, length and width of vessels) is an important basis for ophthalmologists to diagnose and treat diseases. The main structures in a normal retinal fundus image are optic disc, macula, blood vessels, etc., refer to Fig 1a. In contrast, the structures in a diseased fundus image are microaneurysms, hemorrhages, exudates, cotton wool spots, etc. [4], refer to Fig 1b. In clinical trials, the diagnosis of glaucoma is made by calculating the vertical cup-to-disc ratio (CDR) [5]. The CDR is calculated by dividing the vertical cup diameter (VCD) by the vertical disc diameter (VDD). The normal CDR ranges from 0.3 to 0.4, but a larger CDR is indicative of glaucoma or other ophthalmic neurological disease, refer to Fig 1c and 1d. For the task of segmenting the optic disc and optic cup, it focuses on the localization of the boundaries of the optic disc and the optic cup, which is especially critical for determining the value of the CDR. Traditional optic disc and cup segmentation methods rely on the physician’s professional judgment, but the current choice of using machine learning methods to jointly segment optic discs and cups can greatly relieve the physician’s consultation pressure. For example, Jiang et al. [6] proposed to use convolutional neural networks not only to experiment with retinal vessels but also to further validate the effectiveness of their method by jointly segmenting the optic disc and optic cup. However, the method is not currently applied in real life. If machine learning and other methods are applied in the clinic, it will be a good aid for doctors to efficiently segment blood vessels and optic disc visual cups for diagnosing eye diseases. By further observation of fundus images, we can find that the retinal vessels are thin and thick and closely connected to each other, and the low illumination of the images makes it more difficult to observe the vascular structures. In addition, the difference between the vascular area and the background is not obvious and the fundus image is susceptible to uneven illumination and noise interference. All these reasons simultaneously affect the task of retinal vascular segmentation. Therefore, accurate segmentation of fundus images plays an important role in the initial screening, subsequent diagnosis, and treatment of patients’ ocular diseases. In this paper, we focus on the retinal vessel segmentation task by a machine learning approach. In clinical applications, ophthalmologists usually manually segment the retinal vessels to extract information about the lesion. However, manual segmentation is not only tedious and time-consuming but also requires ophthalmologists to be skilled in avoiding errors. With recent advances in deep learning, automatic segmentation techniques have gradually become the mainstream technique for retinal vessel segmentation. Automatic segmentation of retinal vessels helps ophthalmologists to detect ocular diseases and relieves inexperienced ophthalmologists of the stress of diagnosis, which is important for the clinical diagnosis and treatment of ocular diseases.

In the last two decades, a large number of articles have demonstrated the rapid development of segmentation methods for different medical images [7–10]. In the survey report [7, 8], the researchers described in detail about the development of automatic extraction of retinal vascular studies, which provides a valuable resource for people to better study retinal vascular segmentation in the future. It can be seen that the current mainstream retinal segmentation methods are mainly with the help of computer technology. In the survey report [9], the investigators review segmentation methods and techniques for optic disc and cup boundaries that automatically and accurately calculate the geometric parameters of the optic disc and cup to help ophthalmologists and optometrists screen for glaucomatous disease, but not to replace their work. For the segmentation of the optic disc and the optic cup, it makes more sense to segment them jointly as a separate segmentation task than to segment the optic disc or the optic cup separately. In the survey report [10], the research work on semantic segmentation methods based on deep learning is reviewed, and the survey mentions that a large number of newly proposed semantic segmentation methods using deep learning techniques have emerged in recent years with greatly improved performance, which also indicates their significant usefulness for the segmentation task. However, most methods for segmentation of retinal vessels have over-segmentation or mis-segmentation. The main reasons for these occurrences are the complexity of the retinal vessel structure, as well as by different scales of noise or unbalanced illumination within the image, and low image contrast and spatial resolution. Therefore, it is desirable to devise an effective method for the automatic segmentation of retinal vessels. Conventional retinal vessel segmentation methods use default rules for vessels to specify vein regions without reference to manually labelled labels. Traditional retinal vessel segmentation methods include vein tracking [11], matched filtering [12], morphological characteristics [13], multi-scale analysis [14], and model-based algorithms. These methods are also collectively referred to as unsupervised learning methods. For example, by using B-COSFIRE filters [15], Gabor filters [16], and Gaussian filters [17] for retinal vessel segmentation, these methods aim to eliminate undesired intensity variations in images and suppress background structure and noise. However, because of the simplicity of vascular feature encoding methods based on filters and other methods, and their lack of effective supervision information will lead to the extraction of coarse vascular information and poor final image segmentation, which cannot meet the needs of clinical applications. This largely limits the ease of application of these methods in clinical practice. In addition to this, there are methods such as region growth algorithms, EM algorithms with maximum entropy [18], and hybrid active contour models [19] which have been used for the segmentation of fundus images. The unsupervised method described above identifies target vessels by using the intrinsic association between features without training a classifier, and the method is relatively simple to operate requiring less hardware environment for experiments. More importantly, the poor quality of the actual fundus imaging leads to an increased segmentation error rate and may result in undetected unlabeled fine vessels in low contrast images.

Currently, deep convolutional neural networks (CNNs) have successfully broken the bottleneck of traditional hand-based feature extraction methods, in which especially Fully Convolutional Network (FCN) [20], U-Net [21] and their variants are widely used in the field of fundus image segmentation. This class of methods has the ability to capture features from coarse to fine detail, and it has better data processing and robustness, and better segmentation performance. These make it one of the most popular methods for current segmentation tasks. After the emergence of U-Net, there is an increasing number of U-Net improvement networks for medical data segmentation, which improve the performance of vascular segmentation from different entry points. Among them, Feng Z et al. proposed a patch-based fully convolutional neural network, which improves and accelerates the training class balancing loss mainly by local entropy sampling and skip-connected CNN structure [22]. Hu et al. proposed a multiscale CNN architecture with an improved cross-entropy loss function. Their final application of fully connected conditional random fields (CRFs) to obtain segmentation results of retinal vessels [23]. Due to the repetitive stride and pooling operations in the CNN structure, resolution is inevitably lost and the extracted pixels are difficult to refine, making it impossible to accurately segment the target vessel pixels. In order to detect the edges of blood vessels and fine vessel pixels more accurately, we need to design a more effective retinal vessel segmentation network model to achieve accurate segmentation of blood vessels, especially to improve the accurate segmentation of fine vessels. Alom et al. proposed a recurrent convolutional neural network (RCNN) and a recurrent residual convolutional neural network (RRCNN) based on U-Net. Their approach fused the U-Net, residual networks, and the ability of RCNN to represent features to achieve retinal vessel segmentation, skin cancer segmentation, and lung lesion segmentation [24]. As the depth of the network increases, the convolutional network parameter space increases consequently and the optimization problem becomes more difficult. However, the network architecture designed by Alom et al. is able to train a deeper network structure through the residual module in RCNN, which is a good approach for the network to learn deeper features. Jin et al. proposed Deformable U-Net (DUNet) for retinal vessel segmentation mainly for local features of retinal vessels [25], which introduces deformable convolution in the network structure and adaptively adjusts the sensory field by the changes of vessels that are useful for extracting effective features. Wang et al. designed a hard attention network (HAnet) consisted of three decoders, the first one aiming to dynamically analyze the “hard” and “easy” regions of the image, while the other two decoders were responsible for segmenting the “hard” and “easy” regions of the retinal vessels [26]. Atli et al. proposed a model (Sine-Net) which applies upsampling to capture thin vascular features and then downsampling to capture thick vascular features [27]. Wang et al. and Atli et al. used different strategies to focus on different cases of features for the variability of vascular features, which was effective in extracting the corresponding features, and Sine-Net performed well in the vascular segmentation task especially in the specificity index. The above encoder-decoder structured network usually encoder uses cascaded convolution to extract high level semantic representations, but only uses skip connection to concatenate the features of encoder and decoder. This encoding-decoding structure can enhance the recognition of vessel boundary information which is a good choice for retinal vessel segmentation tasks. However, if the actual receptive field of the network is insufficient it will lead to poor segmentation of fine vessels which requires us to design a more efficient network to alleviate this problem.

All of the above methods have progressed well, but the retinal vessel segmentation method using CNN repeatedly steps and pooling operations resulting in inevitable loss of image resolution. In addition, the fundus image itself makes the segmentation task more difficult due to the complex structure of the fundus image which requires a high imaging environment. Fundus images may contain different scales of noise and image illumination imbalance, as well as low image contrast and low spatial resolution, so image preprocessing methods will be the key to efficient training of network models. When the image acquisition conditions are dark or low light, the presence of low contrast conditions in the image will make the image segmentation task more difficult. In recent years, some effective image preprocessing methods have been proposed for image segmentation tasks, such as Multiscale Retinex (MSR) and Multiscale Retinex with Color Restoration (MSRCR) [28] are widely used for visual tasks, and they are both based on Retinex theory [29] have been proven to be effective by several studies. Ke et al. proposed an Enhanced Deep Convolutional Low-light Image Enhancement Network (EDLLIE-Net) to achieve the effect of low-light image enhancement [30], EDLLIE-Net enhances the extraction ability of features by MSR method but does not perform preprocessing operation on the original color image, which may still have an impact on the subsequent segmentation if the representation of features is not enhanced from the root. Further, Liskowski and Krawiec proposed preprocessing of magnified image slices using global contrast normalization and zero-phase whitening [31], which preprocessed the fundus image image background to be completely white while the vascular structures in the image became more visible in black. The zero-phase whitening of the image removes the correlation between the feature signals, which makes the subsequent extraction process of the overall segmentation difficult. We address the fact that the above methods do not yet fully address the differences between different retinal vessel segmentation methods, as well as the problems posed by poor contrast in fundus imaging and different image lesion areas and large amounts of noise. In this paper, we propose an effective framework for retinal vessel segmentation Reverse Fusion Attention Residual Network (RFARN), which achieves fundus image enhancement and retinal vessel segmentation goals. The main work in this paper is as follows:

1. We propose a multiscale retinex with color restoration (MSRCR)-based preprocessing method for fundus retinal images, aiming to improve image quality, enhance vascular regions, and eliminate imaging noise for better segmentation.
2. We propose a model called Reverse Fusion Attention Residual Network (RFARN) for the automatic segmentation of retinal vessels. RFARN is used as a training network model for the subsequent retinal vessel segmentation task, aiming to reduce the feature redundancy between different layers of the network model and improve the quality of retinal vessel segmentation.
3. In the Reverse Fusion Attention Residual Network (RFARN), the Reverse Channel Attention Module (RCAM) effectively captures the lost edge features and residual detail information in the encoding stage using the reverse idea, and Reverse Spatial Attention Module (RSAM) help to recover the underlying feature information lost during upsampling in the encoder and decoder modules for further improving the accuracy of retinal vessel segmentation.

The rest of this paper is organized as follows. Section II describes the MSRCR-based retinal image preprocessing method and the new retinal vessel segmentation network in this paper. Section III describes the details of the experimental implementation, the experimental performance evaluation metrics, and the analysis of the ablation and comparison experiments. In Section IV, we summarize the full text.

## Methods

**Fig 2:**
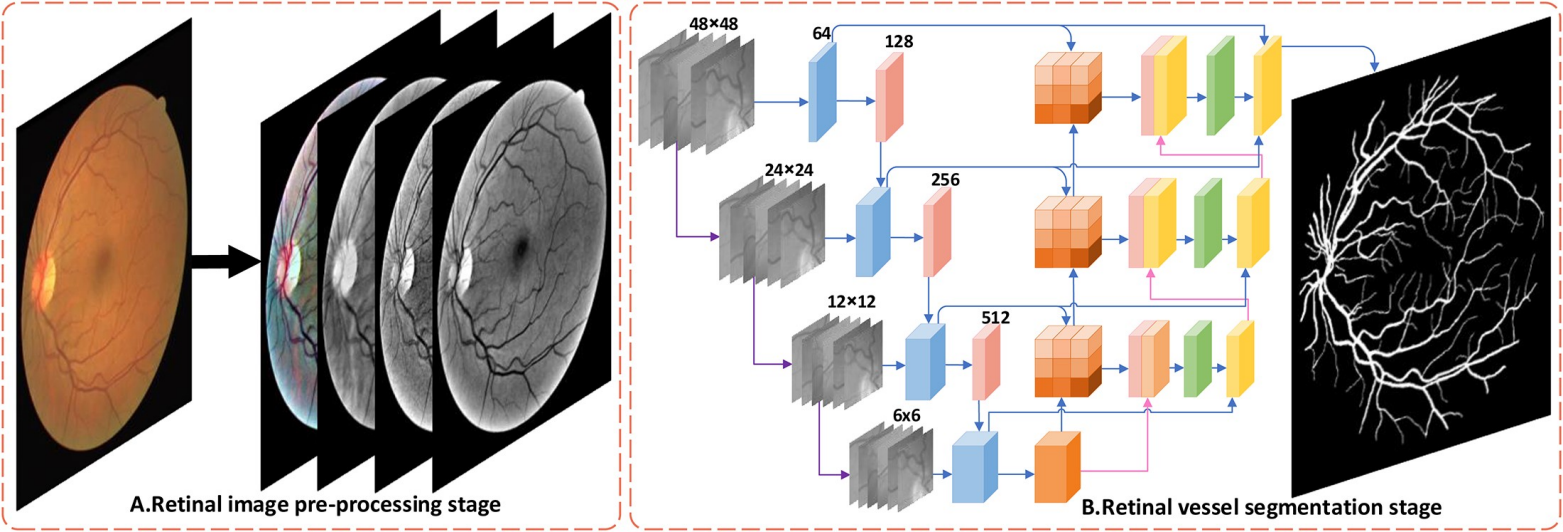
Structure of the proposed retinal vessel segmentation RFARN framework. A is the retinal image pre-processing stage. B is the retinal vessel segmentation stage using the inverse fusion attention residual network.

In this section, we describe the proposed retinal vessel segmentation method RFARN in detail. The new retinal vessel segmentation framework is shown in Fig 2, which consists of two stages, the first phase is retinal image preprocessing and the second phase is retinal vessel segmentation phase.

### Datasets

In the retinal image pre-processing stage, this paper visualizes the results before and after pre-processing on three fundus datasets. In the experimental part of retinal vessel segmentation, this paper validates our proposed method on three fundus datasets. The paper uses the digital retinal image DRIVE [32] for vessel extraction, STARE [33] for retinal structure analysis, and the CHASE (CHASE_DB1) [34] dataset.

The DRIVE dataset is from the Dutch Diabetic Retinopathy Screening Programme. It consists of 40 retinal fundus vascular images with corresponding Ground truth images and corresponding masks images. Of these, 33 images do not show any signs of diabetic retinopathy, while the remaining 7 images show signs of mild early diabetic retinopathy. The size of each image was 565 x 584. (http://www.isi.uu.nl/Research/Databases/DRIVE/).

The STARE dataset consists of 20 retinal fundus images with corresponding hand-labeled images and corresponding mask images. Each image was digitized to 700 × 605. The first 10 images in the dataset are of healthy subjects, while the remaining 10 are images of abnormal pathology with vascular overlap. (https://cecas.clemson.edu/~ahoover/stare/).

The CHASE dataset consists of the left and right retinal images of 14 students and the corresponding Ground truth images and the corresponding mask images. Each image has a resolution of 1280 × 960. Compared to DRIVE and STARE, the images in the CHASE dataset suffer from uneven background illumination, poor contrast of blood vessels, and extensive arterial narrowing. (https://blogs.kingston.ac.uk/retinal/chasedb1/).

### Pre-processing methods for retinal images

**Fig 3:**
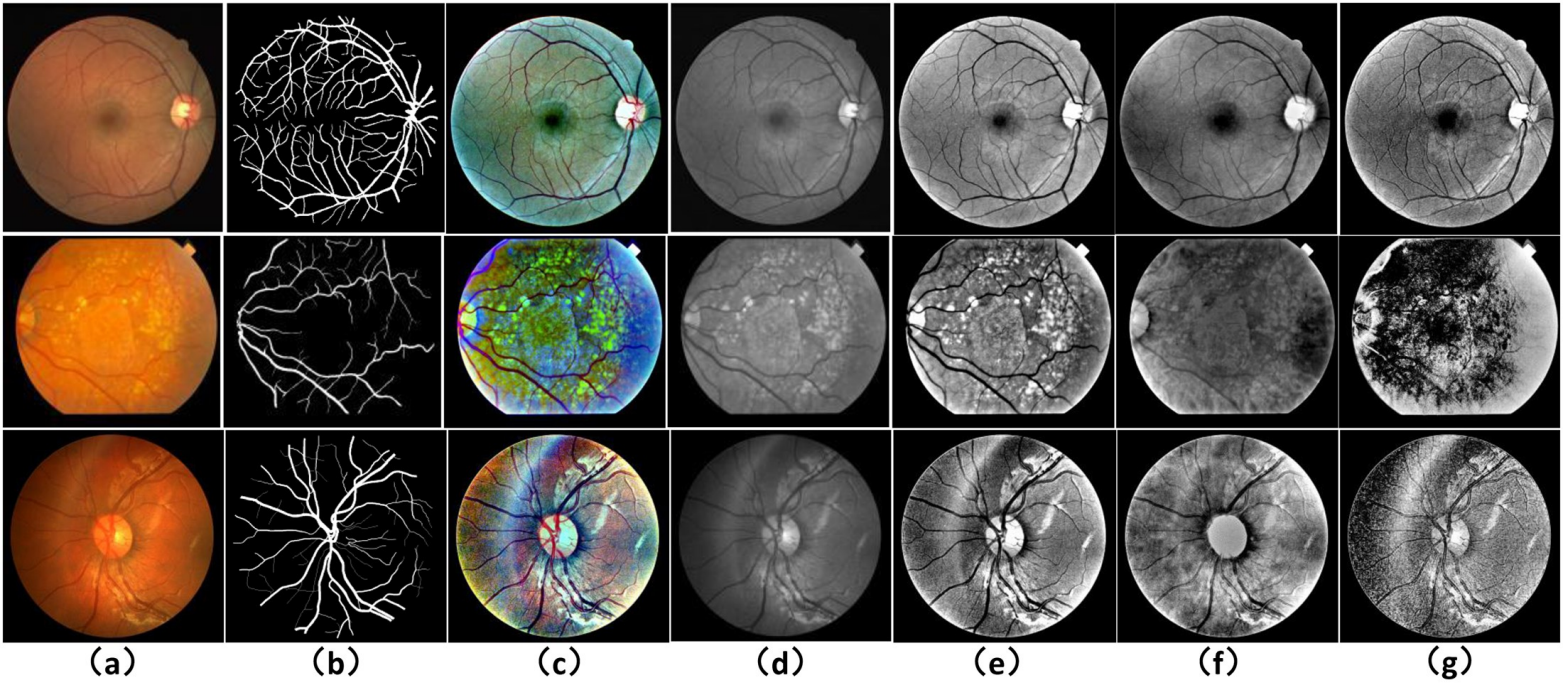
The results of the pre-processing of the retinal images. The first row shows the DRIVE dataset, the second row shows the STARE dataset and the third row shows the CHASE dataset. (a) is the original image of each dataset, (b) is the corresponding Ground truth, (c) is the enhanced retinal image based on the MSRCR method, (d) is the retinal images enhanced by the CLAHE method, (e) is the enhanced G-channel greyscale image, (f) is the enhanced R-channel greyscale image, and (g) is the enhanced B-channel greyscale image.

The diagnosis of ophthalmic diseases requires high-quality fundus images, but the existing fundus imaging is constrained by the imaging environment and imaging equipment, which results in poor quality fundus images and images with low brightness, low contrast and high noise. At the same time, these factors directly create challenges for pathological analysis of fundus images, which may cause analytical errors in severe cases. Therefore, it is essential to improve the visualization of fundus images and to enhance the readability of information and the variability of features in order to obtain more accurate information about the characteristics of blood vessels for pathological analysis. Image enhancement techniques based on Histogram Equalization (HE) [35] are commonly used by researchers, but using balanced histograms to improve the contrast of an image can result in a reduction in the gray level of the image, which in turn results in a loss of image details. To compensate for the shortcomings of HE, Reza proposed the Contrast Limited Adaptive Histogram Equalization (CLAHE) algorithm [36], which largely improves the contrast of images, but the method amplifies background noise and may treat the focal part as a background region, and is not effective in enhancing images with more concentrated gray levels. Therefore, the histogram-based image enhancement method cannot achieve the practical application effect. For the above methods, on the one hand, there is the inability to enhance the overall information of the optic nerve disc, the fundus vessels, and the lesions in the fundus image. On the other hand, most methods directly process color fundus images into grayscale images, which is hardly a real effect of image fidelity. In contrast, the Multiscale Retinex with Color Restoration (MSRCR) image enhancement method based on Retinex theory has been extensively tested on several test scenes and more than one hundred images on multiscale retinas, and solves the grayscale level at the cost of moderate dilution of color consistency defects of the images [28]. Therefore, in this paper, we propose the Multiscale Retinex with Color Restoration (MSRCR) based image preprocessing method, where the fundus images are properly preprocessed so that the network model can learn the information of image features more effectively. The results of pre-processing the DRIVE, STARE and CHASE dataset images are shown in Fig 3. The specific implementation of the retinal image pre-processing method is shown below.

Images are primarily formed by the interaction of incident light and reflected light from an object. Land believes that the color of an object is determined by the object’s ability to reflect long-wave (red), medium-wave (green) and short-wave (blue) light, independent of the incident component [29]. Therefore, the first step of preprocessing is to estimate and remove or reduce the incident component of the original color fundus image by MSRCR method, so as to obtain the reflective component that reflects the essential information of the image and achieve the purpose of image enhancement. Assume that the fundus image *P* is divided into a reflective component *R* and an incident component *I*, as shown in Eq (1):

$$\begin{array}{c} P_{i}\left(x,y\right)=R_{i}\left(x,y\right)\times I_{i}\left(x,y\right), \end{array}$$ (1)

Where *P*_*i*_(*x*, *y*) is the distribution of the fundus image in the *i*-th spectral band, *R*_*i*_(*x*, *y*) is the reflectance component of the Retinex output. The effect due to illumination factors is attenuated by separating the incident component *I* in the fundus image *P*. Therefore, the logarithmic domain processing of Eq (1) achieves the purpose of removing the incident component and enhancing the image detail information. As shown in Eq (2):

$$\begin{array}{c} R_{i}\left(x,y\right)=\text{log}\;P_{i}\left(x,y\right)-\text{log}\;\left(F\left(x,y\right)\times P_{i}\left(x,y\right)\right), \end{array}$$ (2)

Where *F*(*x*, *y*) is a Gaussian surround function. The smaller the value the greater the range of pixel value intensity compression and the more prominent the detail of the image. The larger its value the better the overall effect of the image, the more natural the image color, but the less clear the local detail. To achieve a balance between the local detail information, the color fidelity performance, and the pixel value compression range properties of the image. Firstly, *R*_*i*_(*x*, *y*) at different scales are weighted and averaged to find the sum. Then, the gray-level image is obtained by using the proportional relationship between each channel of the color image, which not only ensures the color information of the fundus image, but also reduces the noise of the image to highlight the detail information. As shown in Eq (3):

$$\begin{array}{c} R_{MSRCR_{i}}\left(x,y\right)=C_{i}\left(x,y\right)\times \sum_{m=1}^{M}W_{m}R_{i}\left(x,y\right), \end{array}$$ (3)

where *C*_*i*_(*x*, *y*) is the color restoration function, *W*_*m*_ is the weighting factor for the different scales, *M* is the number of scales.

The first step in pre-processing has yielded an overall enhancement of the fundus image. Next, we need to focus on the most important vascular structures in the fundus image. Inspired by the fact that single-channel grey-scale images show the contrast between the blood vessels and the background better than RGB images [25]. So the second step of preprocessing converts the RGB fundus image processed by the MSRCR method into a single-channel greyscale image [37]. The conversion is as in Eq (4):

$$\begin{array}{c} I_{gray}=0.299\times R+0.587\times G+0.114\times B, \end{array}$$ (4)

where *R*, *G*, *B* denote the red, green and blue channels respectively. According to the formula, it can be seen that the *R* channel accounts for 29.9% of the converted grey-scale image, the *G* channel accounts for 58.7% and the *B* channel accounts for 11.4%. By decomposing the RGB color fundus image into monochrome images of the red, green, and blue channels, it can be seen that a large number of high luminance set pixels in the *R* channel are concentrated around the optic disc. This results in more blurring of the blood vessels around the optic disc, while the *G* channel pixels are less luminous. However, the pixels around the optic disc in the *G* channel image are clearer, which is important for the model to learn the relevant features of the blood vessels. Further analysis of the *R*, *G* and *B* channel images shows that the *R* and *B* channel images have a lot of noise and low contrast, while the *G* channel images have a high degree of differentiation between the blood vessels and the background. So we used the *G* channel as a base and incorporated the *R* and *B* channels into the *G* channel proportionally. This retains some of the feature information of the *R* and *B* channels while making maximum use of the information of the *G* channel. Finally, the grey-scale image of the *G* channel is fed into the network.

To aid the training of the retinal vessel segmentation network, we normalized the retinal images to improve the convergence speed of the network [38]. Therefore, the third step of preprocessing is to normalize the retinal images to the data. First, we used the Z-score normalization method [39] to dimensionally normalize the dataset, i.e., each dimension of the fundus dataset *I* was set to have zero mean and unit variance. The Z-score normalization transformation was as in Eq (5):

$$\begin{array}{c} I_{norm}=\frac{I-\mu }{\sigma },I\in I_{1},I_{2},\ldots ,I_{n} \end{array}$$ (5)

where *I* ∈ [*I*_1_, *I*_2_, …, *I*_*n*_] denotes the fundus datasets, *μ* is the mean of *I*, and *σ* is the standard deviation of *I*. Since there are positive and negative values of *I* after normalization, and the mean of *I* is 0, the variance is 1. Therefore, we mapped the values of the fundus image data *I* to the range 0-255 by Min-Max normalization as in Eq (6):

$$\begin{array}{c} i_{j}=\frac{i_{j}-i_{min}^{j}}{i_{max}^{j}-i_{min}^{j}}\times 255, \end{array}$$ (6)

where *i*_*j*_ ∈ *I*_*norm*_, *i* ∈ [1, 2, …, *n*]. The above steps are the specific fundus retinal image pre-processing process.

### Reverse fusion attention residual network for retinal vessel segmentation

**Fig 4:**
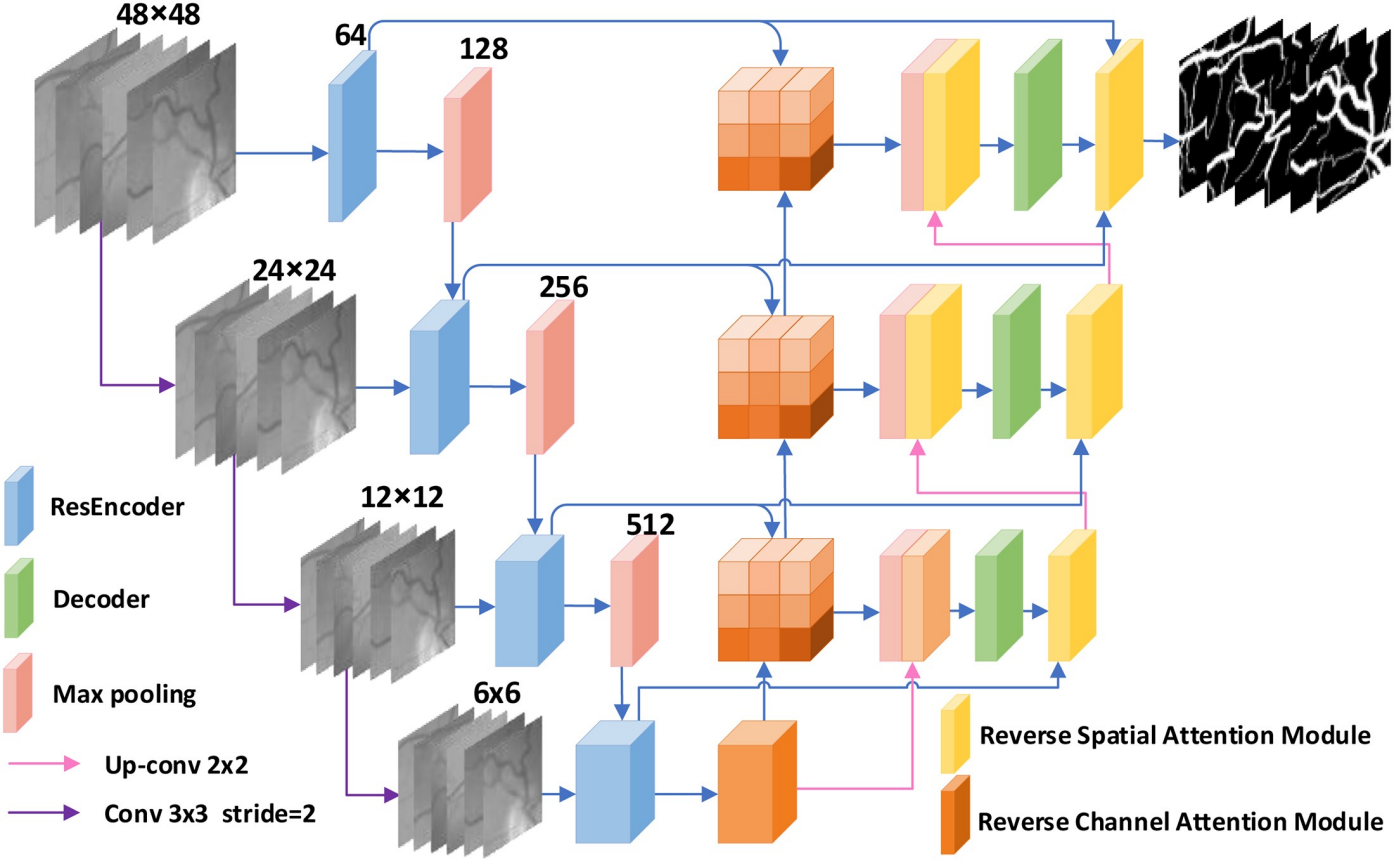
RFARN network structure.

This section describes in detail the Reverse Fusion Attention Residual Network (RFARN) proposed in this paper for retinal vessel segmentation. RFARN is mainly composed of residual encoder module, reverse channel attention module (RCAM), decoder module, and reverse spatial attention module (RSAM). RFARN uses side inputs to construct image pyramids, which fuse different levels of image features to improve the extraction of network feature information. Specifically, the side inputs of RFARN are divided into four branches, each with image sizes of 48 × 48, 24 × 24, 12 × 12, and 6 × 6 pixels, respectively. The specific RFARN network structure is shown in Fig 4.

#### Residual encoder module

**Fig 5:**
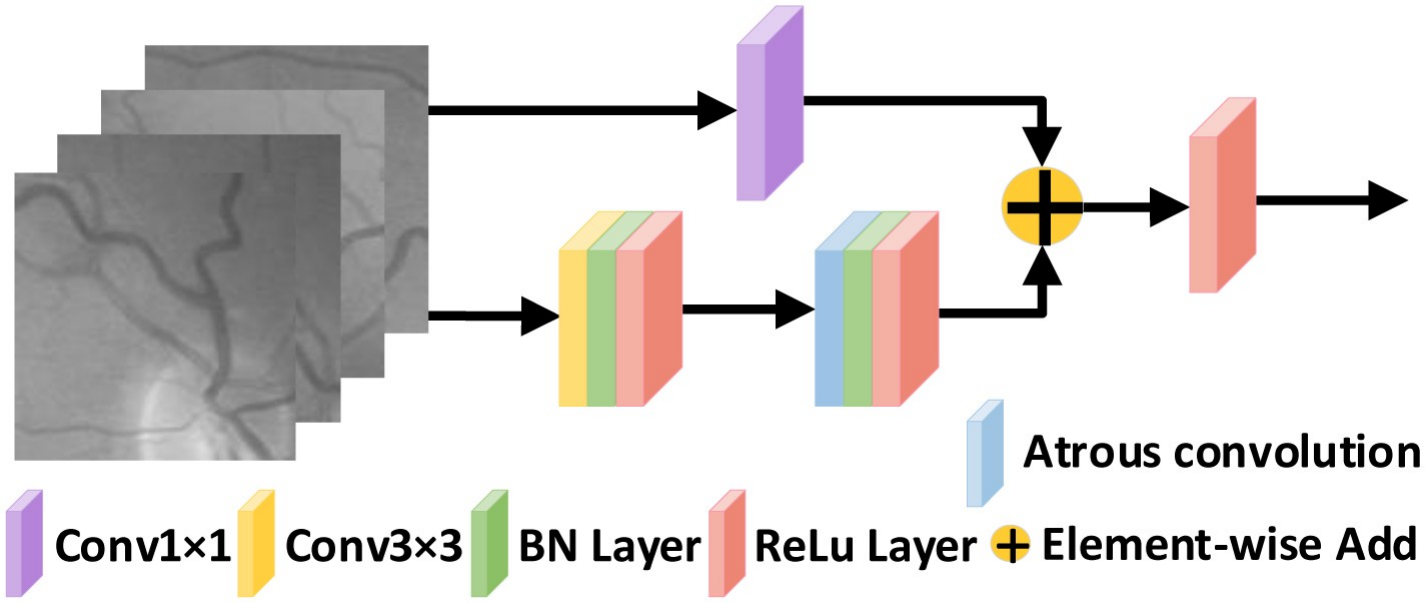
Residual encoder structure.

The introduction of Residual Learning and Identity Mapping by Shortcuts in the network structure makes the deep network easier to optimize and does not produce higher training error rates, and even reduces the segmentation error rate [40]. We improve the encoder module by referring to the residual module in Residual Network, but using the residual module to convolve first and then pool, which causes the loss of semantic information in the image by continuous subsampling and a single convolution kernel size cannot accurately capture the thin and thick vascular features. This problem can be alleviated by atrous convolution [41], which allows a large range of features to be extracted while increasing the perceptual field and preserving the information lost by pooling. The residual encoder in RFARN consists of four main encoders based on the residual module. The output features from each side input are fed sequentially to the encoder module, where they are passed sequentially through a 3 × 3 convolution and an atrous convolution layer with an expansion ratio of 2. The original inputs are then summed by the residual connections to accelerate the convergence of the network and avoid gradient disappearance. At the same time, it filters the background noise of the low-resolution feature mapping and highlights important regions in the fundus image. Finally, the output of the features from the residual encoder is subjected to a maximum pooling operation to expand the perceptual field for better extraction of global features, and the pooled features are sent to the next level of the residual encoder. The structure of the residual encoder is shown in Fig 5.

#### Reverse channel attention module

**Fig 6:**
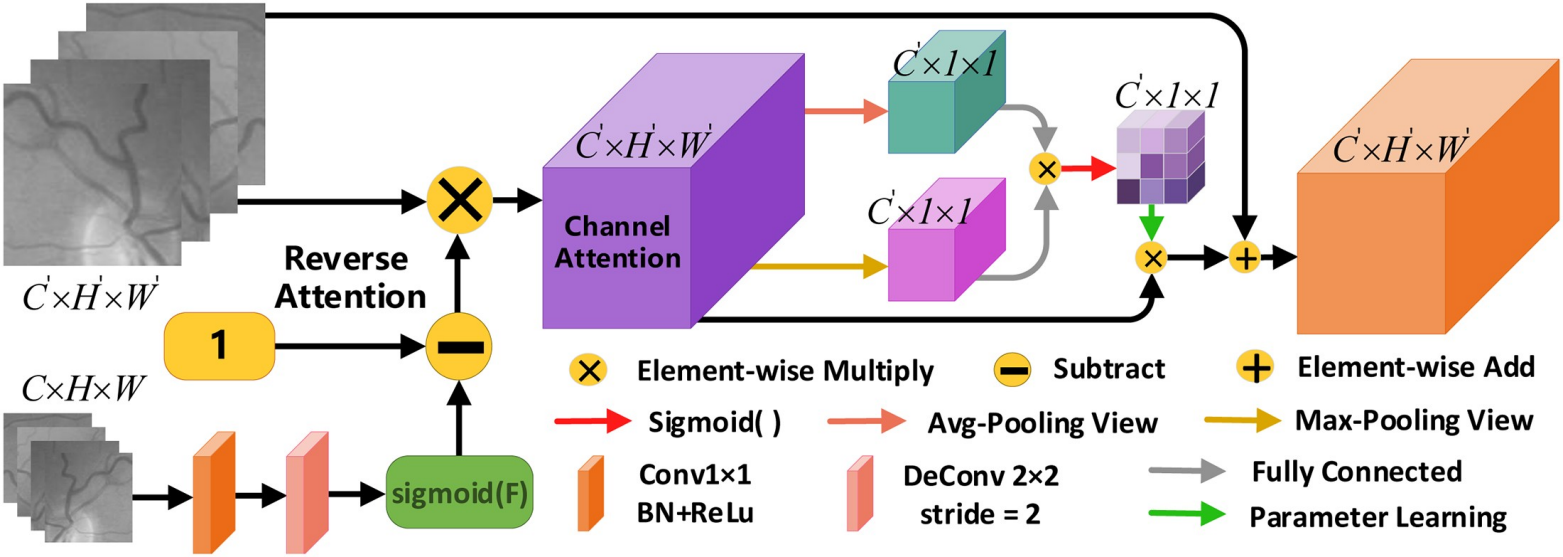
Reverse channel attention module structure.

In the encoding stage, we use atrous convolution to achieve the purpose of expanding the field of perception and capturing more global features. As each downsampling operation results in a loss of edge information of the retinal vessels. In addition, the texture features in the image describe the spatial color distribution and light intensity distribution of small regions of the image. In order to restore as much detailed information as possible, most researchers will take to directly collocate the features of each encoder layer with those of the decoder. However, the vascular features extracted from the shallow encoder module not only contain the edges of the vessels but also retain the texture features of the fundus retinal image. If the loss of these feature information has an impact on the robustness of the higher-level features, it further affects the recovery of the decoder module features [42]. In RFARN, we fuse shallow and deep features by the reverse attention idea [43] as a way to obtain the blood vessel boundary regions in the shallow features and highlight the obscure blood vessel features in the image. For texture information in the image, the interdependent texture features are enhanced and the responsiveness of feature semantics is improved by exploiting the dependencies between different channels [44]. For this purpose, we designed a Reverse Channel Attention Module (RCAM), whose structure is shown in Fig 6. It utilizes the reversal mechanism and the dependencies between different channels to enhance the interdependencies between channel features and improve the feature representation of the feature semantics.

RCAM starts from the feature map generated at the bottom of the RFARN model, which is low resolution but has deep semantic information, so RCAM is used to help the network suppress the currently prominent feature regions to enhance the edges and details in the deep features. First, the feature map *C* × *H* × *W* is converted to 1 × *H* × *W* by a 1 × 1 convolution layer, followed by a deconvolution kernel of 2 × 2 with a stride of 2 to resize the feature map to 1 × *H*′ × *W*′. Then the predicted value generated by the deep feature map 1 × *H*′ × *W*′ is subtracted by 1, so that the high-resolution and complete blood vessel edge regions and details can be obtained, which is implemented as shown in Eq (7):

$$\begin{array}{c} R_{n-1}=1-sigmoid\left(\phi \right)=1-\frac{1}{1+e^{-\phi }}, \end{array}$$ (7)

where *n* denotes the feature generated at the nth residual encoder module, *R*_*n*−1_ denotes the corresponding weight of the (n-1)th residual encoder module. The edge features of the n-1th layer of the residual encoder are obtained by pixel dot product, and the operation is shown in Eq (8):

$$\begin{array}{c} {\hat{R}}_{n-1}=R_{n-1}⊗B_{n-1}, \end{array}$$ (8)

where ${\hat{R}}_{n-1}$ denotes the edge feature of the n-1th layer of the residual encoder, ⊗ denotes the pixel point multiplication, and *B*_*n*−1_ denotes the feature of the (n-1)th layer of the residual encoder. To make the network focus on the edge features of each channel, we input the edge features $\hat{B}\in R^{C^{\prime }\times H^{\prime }\times W^{\prime }}$ into the channel attention. The edge features are reshaped into average pooling features ${\hat{B}}_{Avg}\in R^{N\times C^{\prime }}$ and maximum pooling features ${\hat{B}}_{Max}\in R^{N\times C^{\prime }}$ by average pooling operation and maximum pooling operation. Since the two types of pooling operations are performed on the semantic information of the same channel features. Therefore, we use a fully connected layer to multiply the transpose of the average pooled feature with the maximum pooled feature, and afterwards reshape the attentional feature map *C_B_* ∈ *R*^*C*′×1×1^ that obtains stronger channel correlation strength. This is done as shown in Eq (9):

$$\begin{array}{c} C_{B}\left(i,j\right)=\frac{exp({\hat{B}}_{Avg(i,1,1)}⊗{\hat{B}}_{Max(j,1,1)})}{\sum_{i=1}^{C}exp({\hat{B}}_{Avg(i,1,1)}⊗{\hat{B}}_{Max(j,1,1)})}, \end{array}$$ (9)

where ⊗ denotes pixel point multiplication, *C*_*B*_(*i*, *j*) denotes the similarity between the i-th channel and the j-th channel, *C* denotes the channel element, ${\hat{B}}_{Avg(i,1,1)}$ and ${\hat{B}}_{Max(j,1,1)}$ denote the average pooled feature and the maximum pooled feature respectively. After that, the reshaped channel feature map *C*_*B*_ is activated by sigmoid and the optimal channel attention matrix *A*_*B*_ is obtained by the parametric learning function, and then the channel attention matrix *A*_*B*_ is multiplied with the edge features as the final output of channel attention. As shown in Eq (10):

$$\begin{array}{c} CA_{B}=A_{B}⊗\hat{B}=\rho \left(\delta \left(C_{B}\right)\right)⊗\hat{B}, \end{array}$$ (10)

Where *CA*_*B*_ represents the final channel attention output, *A*_*B*_ represents the optimal channel attention matrix, $\hat{B}$ represents the edge features, *ρ* represents the parametric learning function, *δ* represents the sigmoid function, *C*_*B*_ represents the reshaped channel features, and ⊗ represents the pixel point multiplication. Finally, the channel feature map *C*_*B*_ and edge feature $\hat{B}$ are matrix multiplied and then summed pixel by pixel with the output feature map *B* of the residual encoder module. In this way, RCAM obtains the final output feature map *C*′ × *H*′ × *W*′. Such an operation not only emphasizes the feature mapping associated with the class but also gives a strong global correlation for each channel, which helps to improve the discriminability of the features.

#### Decoder module

**Fig 7:**
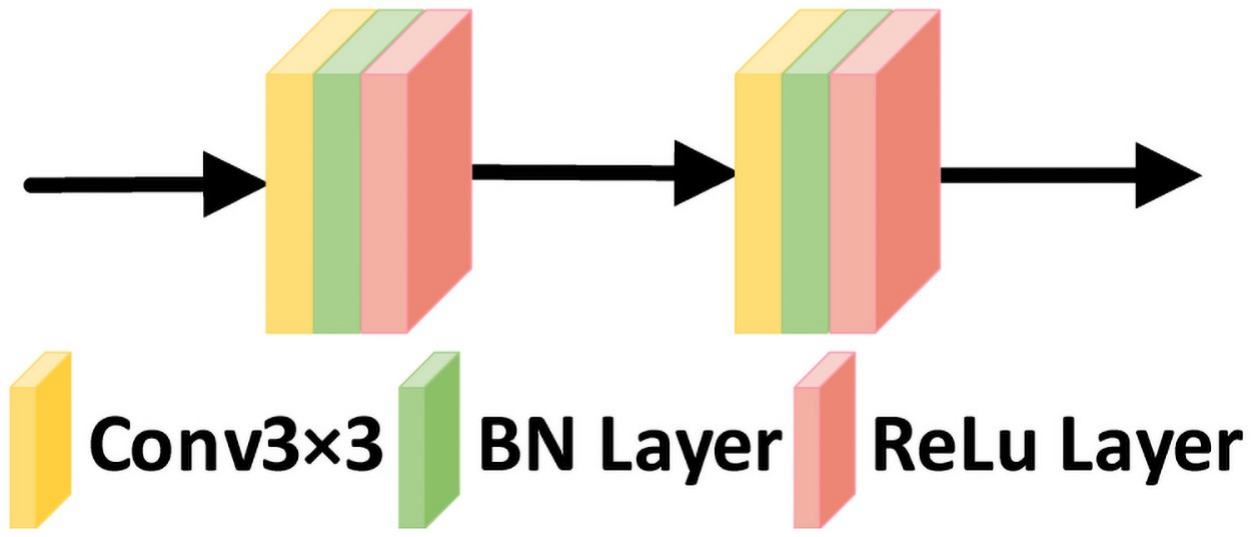
Decoder structure.

During decoding, each decoding stage uses a deconvolution kernel of 2 × 2 with a stride of 2 to recover the feature size, followed by two serial 3 × 3 convolutions to extract the features, and finally ReLU activation is used. To aid the decoding process, the feature maps generated by the reverse channel attention module and the underlying feature maps are stitched together by using skip connections to capture the fine feature information retained in the residual encoder module. In addition, considering the variability in the feature distribution of fundus vascular images in different layers of the network, we send the output of the decoder to the proposed reverse spatial attention module to highlight the local vascular regions of the decoding module output feature maps. The decoder structure is shown in Fig 7.

#### Reverse spatial attention module

**Fig 8:**
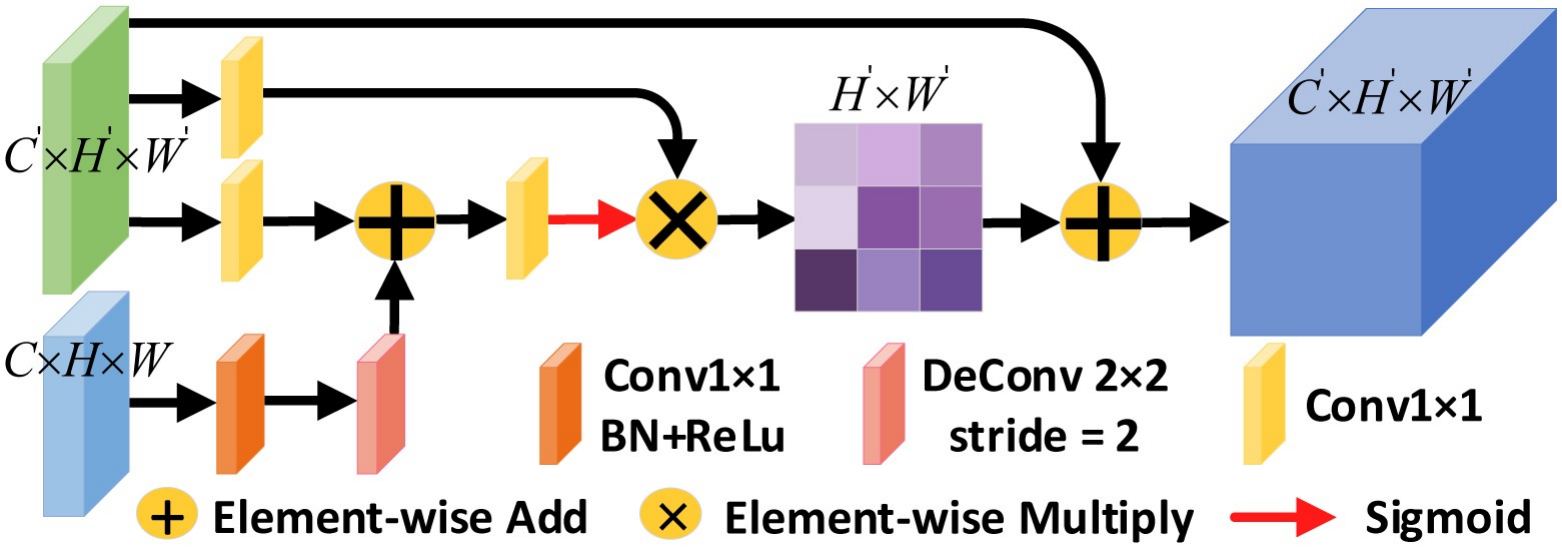
Reverse spatial attention module structure.

Inspired by stacking multiple network models to achieve a horizontal increase in the depth of the network and direct delivery of predicted images to sub-network models for deblurring [45]. We propose to embed the reverse spatial attention modes between different decoding layers to further improve the performance of network recovery features. Firstly, the output of the features from the residual encoder and decoder are used as input to the RSAM. This provides more granular feature information for the current decoding stage to recover the retinal image. Secondly, as the features which are attended to usually come from a lower level, the decision to pay attention is made by the features from a higher level. This means that we need to pay attention to the feature information from the different encoder layers and eventually pass all useful features to the decoder module. Thus, we focus on spatial features between different layers of encoders and decoders employing a cross-layer approach based on the idea of spatial attention. Compared with the simple transfer of the output of the upper level and the addition of the features of the same level, RSAM facilitates the propagation of feature information cross-layer and cross-level. The RSAM structure is shown in Fig 8.

RSAM differs from RCAM in that RCAM emphasizes focusing on the less prominent detail regions in the feature map, while RSAM is targeted at the local features of the current retinal image to achieve better segmentation results. First, RSAM takes the previous stage encoded feature map *C* × *H* × *W* and generates a residual feature 1′ × *H*′ × *W*′ of the same size as the next stage decoded feature map by an upsampling operation. And the corresponding stage decoder output features and the residual features are simultaneously used as the input to RSAM. Next, the decoder feature map *C*′ × *H*′ × *W*′ is fed into two parallel branching operations to extract local features of the feature map using the idea of spatial attention [44], which generates the corresponding attention-guided features. The first branch uses a 1 × 1 convolution and a convolution kernel of 2 × 2 whose stride is 2 to deconvolute the residual features with the features of the previous decoder for element-by-element summation, and the reverse use of the bottom features is for better fusion with the top-level features. In addition, pixel attention masks are generated by using 1 × 1 convolution and sigmoid activation, so that stronger semantic information is extracted for the higher-level features. These masks are multiplied with the decoder feature map *C*′ × *H*′ × *W*′ after 1 × 1 convolution are used to rescale the high-level local features *H*′ × *W*′ as a way to capture the spatial dependence between any two positions of the feature map. This is done as shown in Eq (11):

$$\begin{array}{c} S_{B}\left(x,y\right)=\frac{exp(R_{x}\times D_{y})}{\sum_{x=1}^{N}exp(R_{x}\times D_{y})}, \end{array}$$ (11)

where *S*_*B*_(*x*, *y*) denotes the similarity between the x-th channel and the y-th location, *R*_*x*_ denotes the inverse pixel feature generated after 1 × 1 convolution and sigmoid activation, and *D*_*x*_ denotes the decoder feature after 1 × 1 convolution. Finally, the new attention-enhanced features *S*_*B*_ are summed with the decoder feature map *C*′ × *H*′ × *W*′ and passed to the decoder module at the next stage. The attention features generated by RSAM suppress the features that are less informative in the current phase, and it allows only valid features to be passed to the next decoding phase, which helps to improve the segmentation performance of the network.

## Ablation experiments

This section details the implementation of the method proposed in this paper for the retinal vessel segmentation task on the DRIVE, STARE and CHASE datasets. It also discusses the analysis of experimental results and visualization results of retinal vessel segmentation.

### Implementation details

The RFARN proposed in this paper is based on the deep learning open source framework Pytorch [46], Linux operating system, Intel(R) Xeon(R) Gold 5218 2.30GHz CPU, and Quardro RTX 6000 24G GPU with 187G of running memory. The programming language used to build the network model is Python 3.7, and the main library packages used are Pytorch 1.4, OpenCV 4.1.2, Numpy 1.18.1, etc. In the training phase, gradient descent was performed using the Adam [47] optimiser with parameters set by default to *β*_1_ = 0.9, *β*_2_ = 0.999 and *ϵ* = 1e-8. The learning rate was attenuated by Plateau’s method [48], with learning rate *lr* initialized to 0.001 and weight attenuation coefficient of 0.0005. To prevent the risk of overfitting and to improve model performance, this paper adopts random dynamic extraction of small batches of patches to train the network. RFARN randomly extracted image patches of size 48 × 48 on the DRIVE, STARE and CHASE datasets with a batch initialization of 32 and a training period of 200. The probability threshold in the three standard data sets was set to 0.49. The RFARN model uses a cross-entropy loss function. The definition is given in Eq (12):

$$\begin{array}{c} L\left(p_{i},q_{i}\right)=-\frac{1}{n}\sum_{i}\left[p_{i}\;\text{log}\;q_{i}+\left(1-p_{i}\right)\cdot \text{log}\;(1-q_{i})\right] \end{array}$$ (12)

Where *p*_*i*_ denotes the true label and *q*_*i*_ denotes the predicted image.

In this paper, DRIVE, STARE and CHASE fundus image datasets are used for experiments. For the DRIVE dataset, 20 images were used for training and 20 images were used for testing. For the CHASE dataset, 20 images are used for training and 8 images are used for testing [49]. For the STARE dataset, the “leave-one-out” method was used for training [25, 50, 51] since there were only 20 images in total and there was no division between the training and testing sets. Nineteen images were selected as the training set and the remaining one image was used for testing. The process was repeated by changing the test images until all images in the dataset were used for testing once.

In the test phase, each test image of each dataset is sequentially extracted with image patches in a sliding window with a sliding step of 5 pixels, and the part of the sliding window beyond the image is filled with zeros.

### Performance evaluation indicators

To evaluate the effectiveness of this method and other existing methods for retinal vessel segmentation, we used five commonly used metrics to objectively assess the segmentation performance of retinal vessels, including Accuracy, Sensitivity, Specificity, ROC curve area, and *F*1_*score*_, each calculated as in Eqs (13)–(18):

$$\begin{array}{c} Accuracy=\frac{TP+TN}{TP+FP+TN+FN} \end{array}$$ (13)

$$\begin{array}{c} Sesitivity=\frac{TP}{TP+FN} \end{array}$$ (14)

$$\begin{array}{c} Specificity=\frac{TN}{TN+FP} \end{array}$$ (15)

$$\begin{array}{c} Precision=\frac{TP}{TP+FP} \end{array}$$ (16)

$$\begin{array}{c} Recall=\frac{TP}{TP+FN} \end{array}$$ (17)

$$\begin{array}{c} F1_{score}=2\times \frac{Precision\times Recall}{Precision+Recall} \end{array}$$ (18)

Where *TP* is the number of vascular pixels correctly segmented, *FP* is the number of vascular pixels incorrectly segmented as background pixels, *TN* is the number of background pixels correctly segmented and *FN* is the number of background pixels incorrectly segmented as vascular pixels.

### Evaluating performance before and after image enhancement and model improvement

We experimentally validated the effectiveness of the retinal image pre-processing method MSRCR and the Reverse Channel Attention Module (RCAM) and Reverse Spatial Attention Module (RSAM) in the Retinal Vessel Segmentation Network RFARN proposed in this paper. Under the same experimental environment settings, this paper uses ResUNet as the baseline network, and conducts retinal vessel segmentation experiments in the DRIVE, STARE and CHASE datasets. For the implementation details of the experiment, the effectiveness of each module in RFARN is verified by ablation experiments. For the implementation details of the experiment, the effectiveness of each module in RFARN is verified by ablation experiments. Specifically, the ablation experiments in this paper were conducted based on ResUNet sequentially fusing the residual encoder module, the multi-scale Retinex color recovery based retinal image preprocessing method, the Reverse Channel Attention Module (RCAM), and the Reverse Spatial Attention Module (RSAM). The experimental results for the model variations of the DRIVE, STARE and CHASE datasets are shown in Tables 1–4. In the table, ResUNet denotes UNet with side input section and residual encoder module, MSRCR denotes multiscale retinex with color restoration based preprocessing method for retinal images, RCAM denotes reverse channel attention module, RSAM denotes the reverse spatial attention module, and RFARN denotes Reverse Fusion Attention Residual Network using MSRCR retinal image preprocessing method. The data in the table are in the format of mean/standard deviation.

**Table 1:** Comparison of experimental results for DRIVE dataset model improvement.

Methods	Acc	Se	Sp	AUC	F1
*ResUNet*	0.9650/0.0040	0.7947/0.0655	0.9717/0.0034	0.9842/0.0040	0.8023/0.0332
*ResUNet* + *MSRCR*	0.9697/0.0029	0.8707/0.0498	0.9784/0.0035	0.9903/0.0032	0.8284/0.0164
*ResUNet* + *MSRCR* + *RCAM*	0.9700/0.0032	0.8782/0.0517	0.9790/0.0034	0.9903/0.0031	0.8395/0.0184
*ResUNet* + *MSRCR* + *RSAM*	0.9703/0.0030	0.8745/0.0518	0.9797/0.0035	0.9904/0.0031	0.8403/0.0179
*RFARN*(*ours*)	**0.9712 / 0.0029**	**0.8788 / 0.0498**	**0.9803 / 0.0034**	**0.9910 / 0.0030**	**0.8453 / 0.0163**

**Table 2:** Comparison of experimental results of DRIVE dataset pre-processing.

Methods	Acc	Se	Sp	AUC	F1
*ResUNet* + *CLAHE*	0.9683	0.8081	**0.9836**	0.9839	0.8168
*ResUNet* + *MSRCR*	0.9697	0.8707	0.9784	0.9903	0.8284
*Khan*(*GLM*)	0.9600	0.7470	0.9800	0.9658	-
*Khawaja*(*CLAHE*)	0.9561	0.8027	0.9733	-	-
*Khawaja*(*GLM*)	0.9603	0.7907	0.9790	-	-
*RFARN*(*MSRCR*)	**0.9712**	**0.8788**	0.9803	**0.9910**	**0.8453**

**Table 3:** Comparison of experimental results for STARE dataset model improvement.

Methods	Acc	Se	Sp	AUC	F1
*ResUNet*	0.9774/0.0033	0.8193/0.1040	0.9831/0.0045	0.9732/0.0057	0.8232/0.0358
*ResUNet* + *MSRCR*	0.9788/0.0027	0.8479/0.0474	0.9828/0.0032	0.9864/0.0040	0.8386/0.0228
*ResUNet* + *MSRCR* + *RCAM*	0.9791/0.0035	0.8467/0.0443	0.9886/0.0043	0.9936/0.0015	0.8577/0.0263
*ResUNet* + *MSRCR* + *RSAM*	0.9795/0.0042	0.8514/0.0362	0.9866/0.0040	0.9942/0.0043	0.8687/0.0262
*RFARN*(*ours*)	**0.9822 / 0.0031**	**0.8874 / 0.0267**	**0.9891 / 0.0023**	**0.9952 / 0.0009**	**0.8707 / 0.0264**

**Table 4:** Comparison of experimental results for CHASE dataset model improvement.

Methods	Acc	Se	Sp	AUC	F1
*ResUNet*	0.9723/0.0040	0.8036/0.0577	0.9839/0.0029	0.9848/0.0040	0.7943/0.0248
*ResUNet* + *MSRCR*	0.9775/0.0038	0.8259/0.0354	0.9889/0.0021	0.9903/0.0027	0.8049/0.0194
*ResUNet* + *MSRCR* + *RCAM*	0.9774/0.0036	0.8243/0.0392	0.9888/0.0020	**0.9905 / 0.0027**	0.8146/0.0184
*ResUNet* + *MSRCR* + *RSAM*	0.9772/0.0037	0.8309/0.0363	0.9883/0.0022	0.9904/0.0026	0.8159/0.0193
*RFARN*(*ours*)	**0.9780 / 0.0035**	**0.8352 / 0.0342**	**0.9890 / 0.0020**	0.9904/0.0027	**0.8185 / 0.0178**

Table 1 shows the results of the self-comparison experiments of the models on the DRIVE dataset. The bolded data in the table indicate the maximum values of the same evaluation metrics for different network models. First, this paper demonstrates the enhancement effect of the MSRCR method on the retinal images of DRIVE, STARE and CHASE datasets from theoretical and visualization perspectives in the retinal image preprocessing section. From Fig 3, it can be observed that the enhanced retinal images of the three datasets images have higher contrast than the original images in terms of blood vessels, noise, and background areas. Observing the smaller blood vessels in the original images, we can find that the proposed preprocessing method successfully enhances their structural features, which can bring significant help to the subsequent segmentation. To further verify the effectiveness of the MSRCR retinal image preprocessing method for vessel segmentation, retinal vessel segmentation experiments were conducted for the ResUNet model and the MSRCR retinal image preprocessing method-based ResUNet model, respectively. From the experimental results in the first and second rows of Table 1, it can be seen that when the MSRCR retinal image preprocessing method is added to the model, the results of all indexes are significantly higher than those of the baseline model, in which the *F*1_*score*_ is increased by 2.61% and the sensitivity is increased by 7.6% which also indicates that the MSRCR retinal image preprocessing method can help the network model to extract the blood vessels which are not captured by ResUNet. From the experimental results, adding the MSRCR preprocessing method improved the sensitivity of the network to the vascular region. After that, to verify the ability of the reverse channel attention module RCAM and the reverse spatial attention module RSAM to capture the boundary and local features of fine vessels of retinal images, we embed the RCAM and RSAM modules respectively based on the ResUNet method of MSRCR retinal image preprocessing method for experiments, as shown in the third and fourth rows of Table 1. From the experimental data in the table, it can be seen that the models incorporating RCAM and RSAM have higher accuracy and *F*1_*score*_ than the baseline model ResUNet, respectively. From the experimental results, the network incorporating RCAM and RSAM modules can learn features better. RCAM enables the network to fuse the vascular edge features valid in the encoder stage with those in the decoder stage, which alleviates the problem of loss of vascular information due to continuous downsampling. In addition, the RSAM module can effectively improve the recovery of local vascular features during upsampling in the decoding stage. Finally, the RCAM and RSAM modules were added together to the ResUNet model with the MSRCR retinal image preprocessing method. Comparing the experimental results of RFARN with the baseline model ResUNet, it was found that the former showed a significant improvement in *F*1_*score*_ and sensitivity, including a 4.3% increase in *F*1_*score*_, 8.41% increase in sensitivity, 0.86% increase in specificity, 0.68% increase in AUC, and 0.62% increase in accuracy.

**Fig 9:**
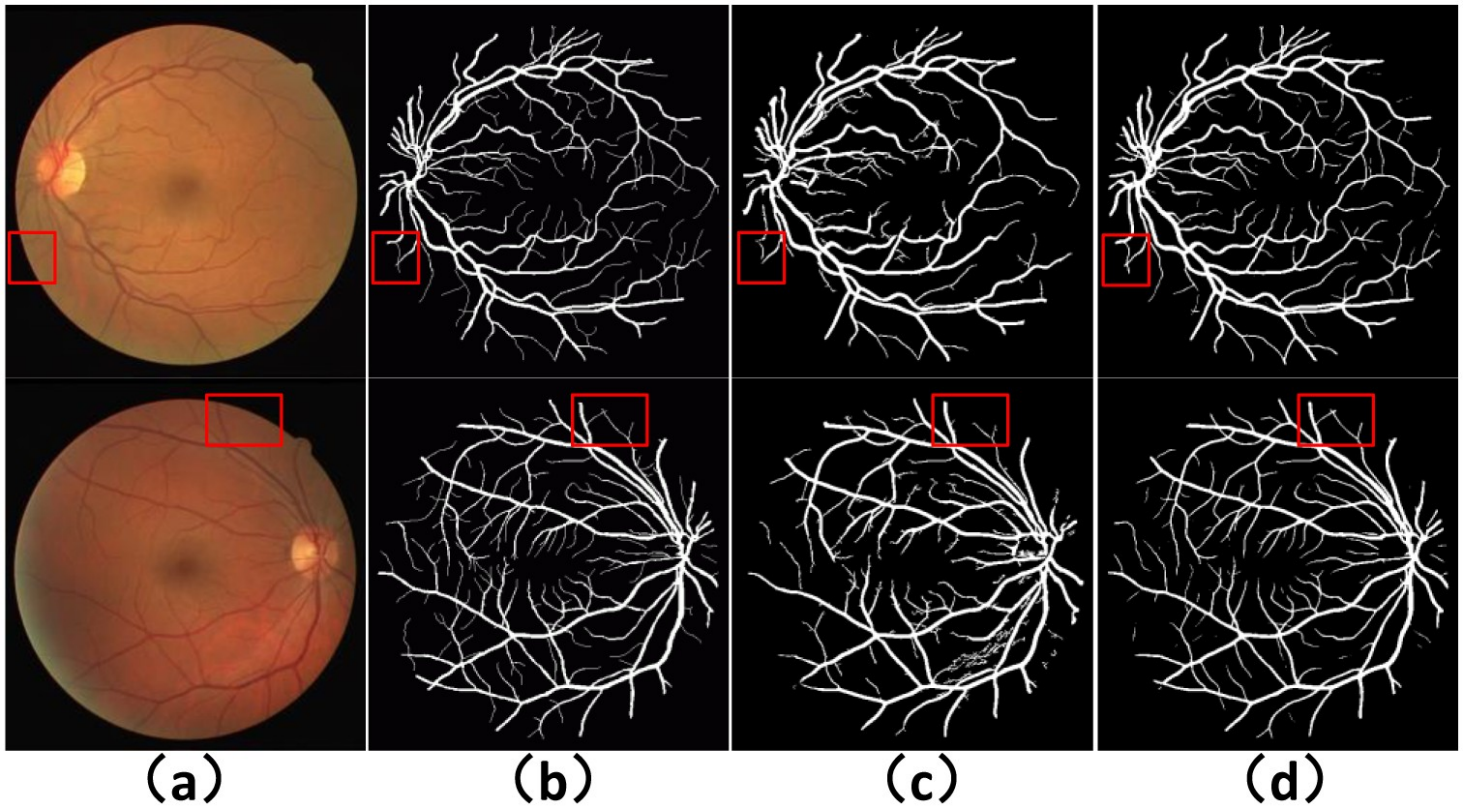
Visualization of segmentation results after different pre-processing in DRIVE dataset. (a) column shows the original image, (b) Ground Truth, (c) column shows the segmentation result based on GLM, and (d) column shows the segmentation result based on MSRCR.

**Fig 10:**
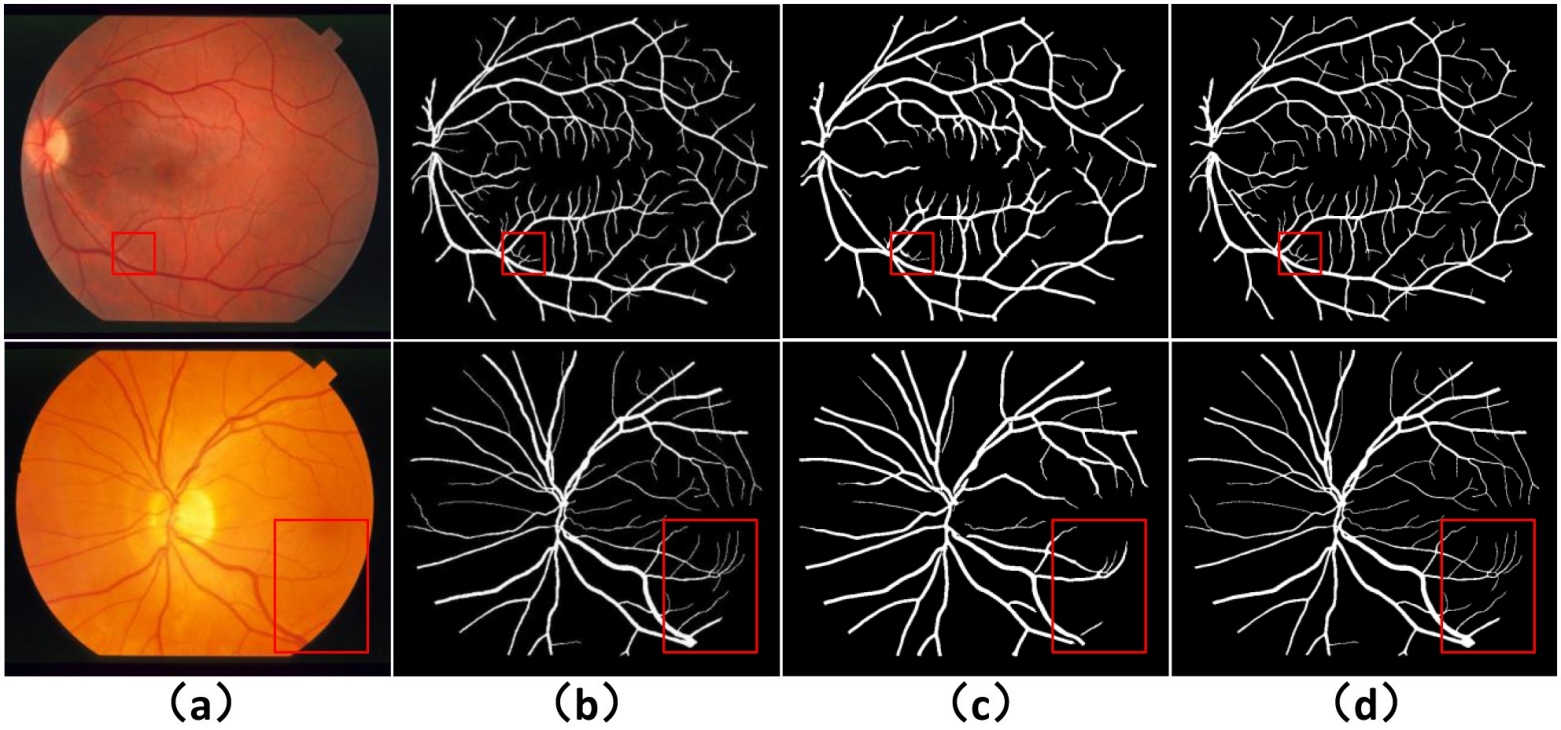
Visualization of segmentation results after different pre-processing in STARE dataset. (a) column shows the original image, (b) Ground Truth, (c) column shows the segmentation result based on GLM, and (d) column shows the segmentation result based on MSRCR.

To validate the retinal image preprocessing method MSRCR proposed in this paper, we compared the segmentation performance with the CLAHE-based preprocessing method experimentally on the DRIVE dataset. In addition to this, we further compare with the Generalized Linear Model (GLM) based retinal vessel segmentation method, where GLM regression is mainly used for non-uniform contrast enhancement. Among them, Khan et al. proposed to use the results of nonuniform contrast enhancement by Generalized Linear Model (GLM) as input [52], and then enhance the vessel features by Frangi filter. Finally, they apply post-processing is applied to eliminate unconnected pixels, which provides quality assurance for the final obtained binary image. Khawaja et al. experimented their proposed segmentation method on two preprocessing models, i.e., on both CLAHE and GLM preprocessing methods yielding competitive results [53]. The specific experimental results are shown in Table 2. We can see by the experimental results in the first and second rows that MSRCR performs somewhat better than CLAHE in all metrics. Comparing the results of RFARN, Khan (GLM) and Khawaja (GLM), the segmentation performance of RFARN has improved significantly in terms of sensitivity metrics. In order to compare with GLM, which is currently an advanced preprocessing method, we show the difference between the MSRCR-based method and the GLM-based segmentation method on the DRIVE and STARE datasets by visualization in Figs 9 and 10. The red box in the figure shows that the GLM-based segmentation method suffers from incomplete segmentation and segmentation breakage.

Table 3 shows the comparison experimental results of each module combination on the STARE dataset. On the STARE dataset, RFARN has a good preprocessing method as the foundation, which makes the final segmentation results closer to the real labels. The network with the addition of RCAM and RSAM modules strengthens the response between the vascular features of retinal images, which also helps RFARN to have a stronger learning ability. Compared with the baseline network ResUNet, RFARN has improved all metrics, especially the sensitivity has improved by 6.81% and the *F*1_*score*_ has improved by 4.75%. Among them, the improvement of sensitivity also indicates the increasing responsiveness of RFARN to blood vessels.

Table 4 shows the results of the comparison experiments on the CHASE dataset for each combination of modules. On the CHASE dataset, the difficulty of segmentation is greatly increased by the non-uniform background illumination and low contrast of the blood vessels in the CHASE original images. Therefore, RFARN using MSRCR method to enhance the original image of CHASE can not only alleviate the large arterial stenosis and low contrast in the image but also create a good prerequisite for vessel segmentation. From the experimental results in Table 4, it can be seen that the MSRCR retinal image preprocessing method based on the ResUNet model has better experimental results than the baseline model ResUNet. The detailed features in vascular images cannot be captured by pre-processing methods, so the channel response between features is enhanced by RCAM and the spatial association between features is enhanced by RSAM, which makes the network significantly more sensitive to features. From the table, we can find that RFARN gives better segmentation results than the single introduction of RCAM or RSAM. Compared with the baseline network ResUNet, the sensitivity of RFARN is improved by 3.16% and the *F*1_*score*_ is improved by 2.42%. The comparison of the experimental results from RFARN with the model introducing RCAM or RSAM reveals that RFARN has better segmentation results, which further indicates the effectiveness of the RFARN method for retinal vessel segmentation.

**Fig 11:**
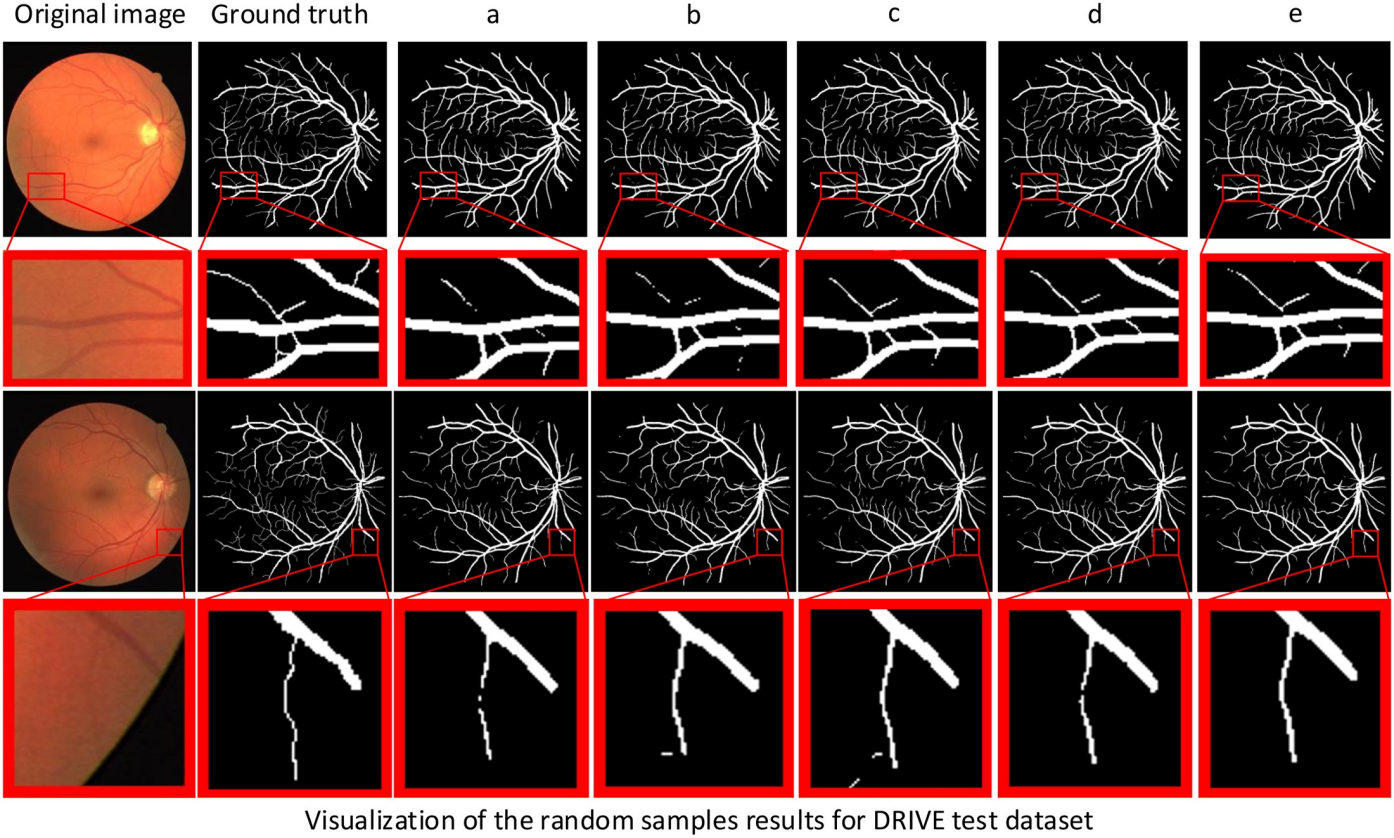
Visualization of segmentation results of different models for random samples on DRIVE dataset. The first column is the original image, the second column is Ground Truth, a column is the ResUNet model, b column is the ResUNet+MSRCR model, c column is the ResUNet+MSRCR+RCAM model, d column is the ResUNet+MSRCR+RSAM model, and e column is the RFARN model.

**Fig 12:**
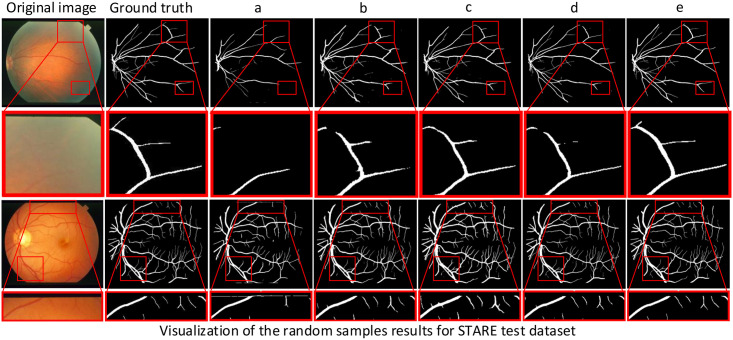
Visualization of segmentation results of different models for random samples on STARE dataset. The first column is the original image, the second column is Ground Truth, a column is the ResUNet model, b column is the ResUNet+MSRCR model, c column is the ResUNet+MSRCR+RCAM model, d column is the ResUNet+MSRCR+RSAM model, and e column is the RFARN model.

**Fig 13:**
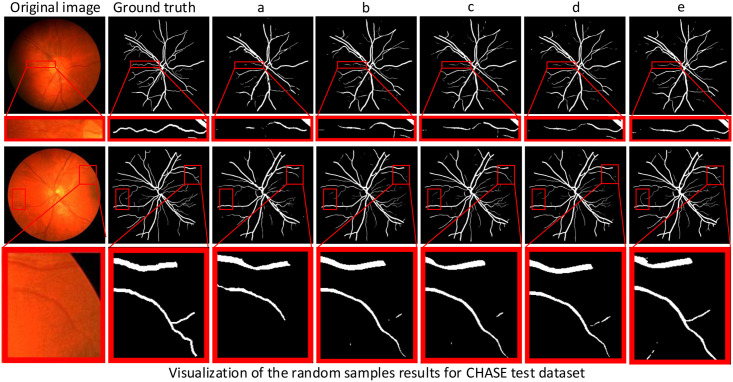
Visualization of segmentation results of different models for random samples on CHASE dataset. The first column is the original image, the second column is Ground Truth, a column is the ResUNet model, b column is the ResUNet+MSRCR model, c column is the ResUNet+MSRCR+RCAM model, d column is the ResUNet+MSRCR+RSAM model, and e column is the RFARN model.

In this paper, we not only perform ablation experiments on different datasets but also visualize the retinal vessel segmentation results of these ablation experiments to illustrate the advantages of RFARN by visualizing the comparison of vessel segmentation. Figs 11–13 show the visualization results of retinal vessel segmentation for each ablation experiment model on the DRIVE, STARE and CHASE datasets. As can be seen from the partially enlarged area in the figure, RFARN shows better segmentation results than the other ablation experiments. In more detail, the ResUNet+MSRCR model shows better segmentation results for the retinal images as a whole, with a significant improvement in vascular continuity. In addition, RFARN has a much better recovery of lost fine vessels and the entire segmented target region than ResUNet’s segmentation visualization. When the RCAM or RSAM module is introduced alone, it can be seen from the red box of the figure that the model handles the connection between the main and terminal vessels better, which indicates that the RCAM or RSAM module can achieve enhanced response to the vascular features of the retinal images. With the inclusion of both the RCAM and RSAM modules, RFARN’s vascular segmentation results are closer to the true labels. Importantly, RFARN has a restorative effect on breaks in fine vessels and is more sensitive to fine vessels. This allows the network to retain more details of the blood vessels when extracting features.

**Fig 14:**
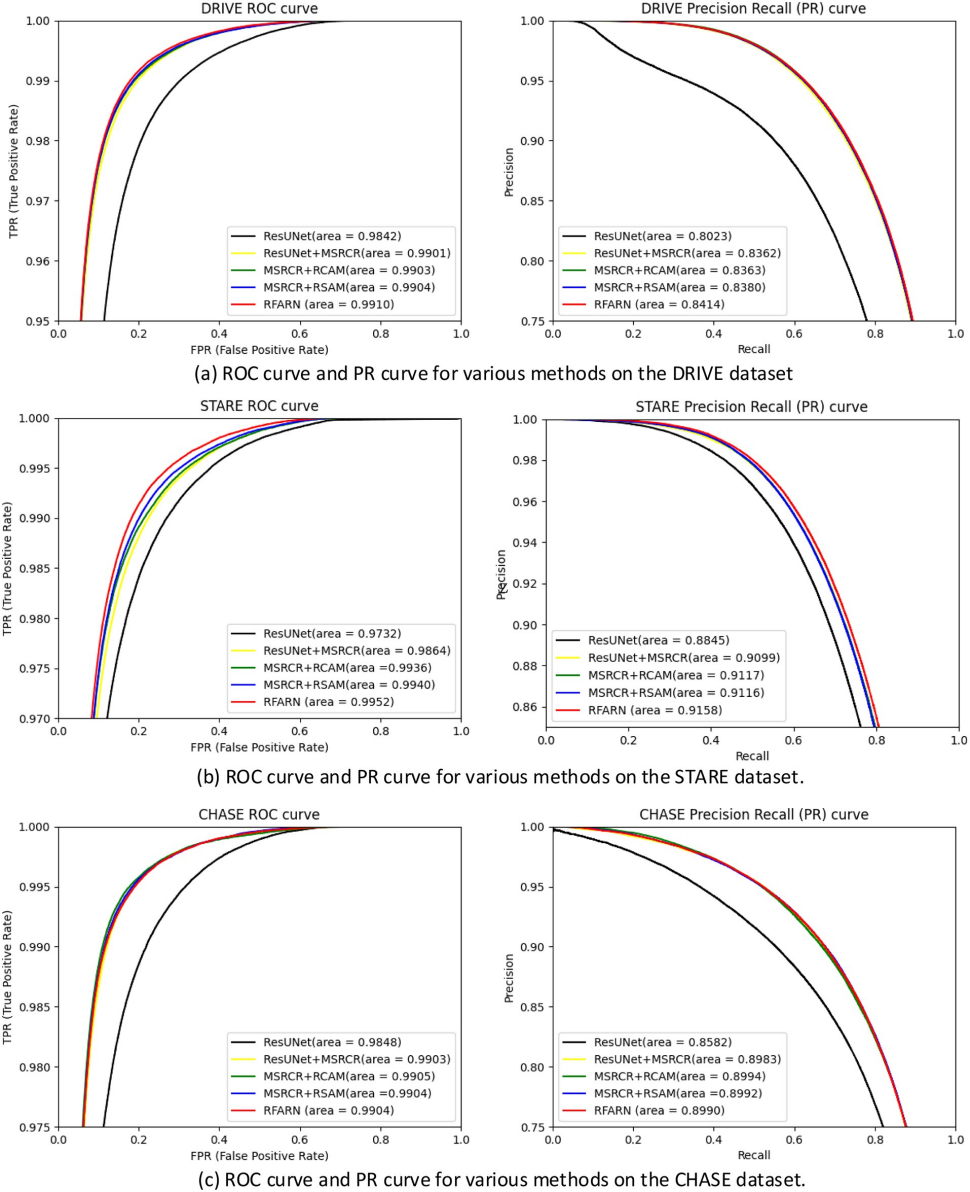
ROC and PR curves for different retinal datasets. (a) ROC curve and PR plot for each model in the DRIVE dataset; (b) ROC curve and PR plot for each model in the STARE dataset; (c) ROC curve and PR plot for each model in the CHASE dataset.

In order to compare the results of the different models in the DRIVE, STARE and CHASE datasets, we have visualized the ROC (Receiver Operating Characteristic) plots and PR (Precision Recall) plots of the above five models. These are shown in Fig 14. The larger the value of AUC, the more efficient the model and algorithm. The experimental results show that RFARN has higher AUC values than the other models for both ROC and PR. The AUC values for ROC on the DRIVE, STARE and CHASE datasets were 0.9910, 0.9952 and 0.9904, respectively, and the AUC values for PR were 0.8414, 0.9158 and 0.8990, respectively. When comparing the plots of RFARN with other models, it was found that the plots of RFARN were all increasing at a positive rate, which further indicated that RFARN outperformed other models in retinal vessel segmentation and was more powerful in preserving retinal image details.

## Discussion

**Fig 15:**
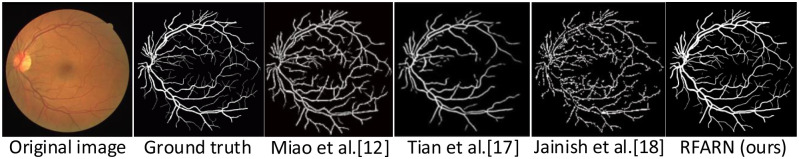
Visualization comparison of unsupervised retinal vessel segmentation results on the DRIVE dataset.

**Fig 16:**
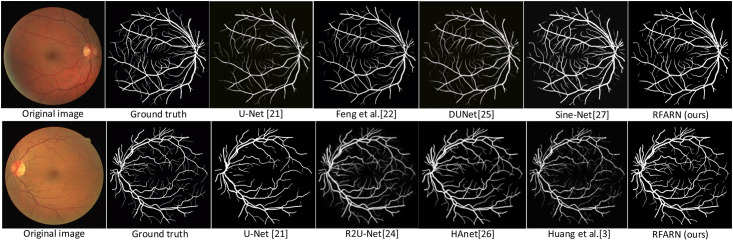
Visualization comparison of the results of supervised retinal vessel segmentation on the DRIVE dataset.

**Fig 17:**
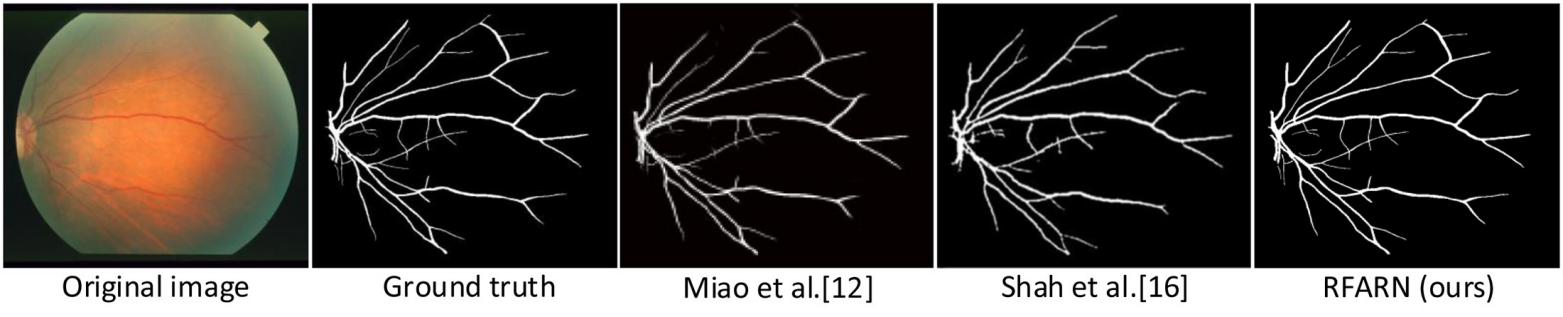
Visualization comparison of unsupervised retinal vessel segmentation results on the STARE dataset.

**Fig 18:**
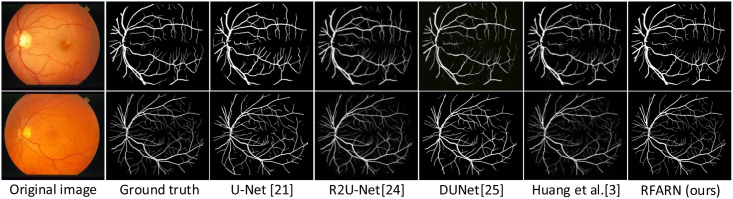
Visualization comparison of the results of supervised retinal vessel segmentation on the STARE dataset.

**Fig 19:**
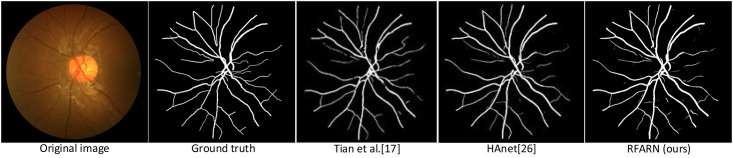
Visualization comparison of unsupervised retinal vessel segmentation results on the CHASE dataset.

**Fig 20:**
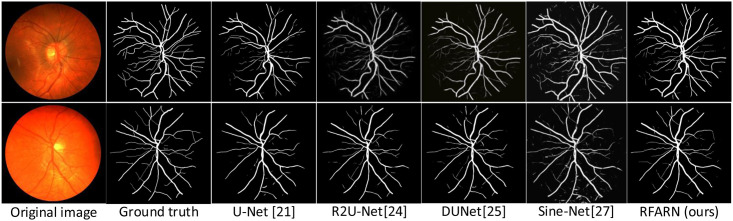
Visualization comparison of the results of supervised retinal vessel segmentation on the CHASE dataset.

In recent years, many network models have achieved good segmentation results in retinal vessel segmentation tasks. The RFARN model proposed in this paper is paved with good pre-processing methods and the improved attention module has also greatly improved the network’s ability to extract vascular features. To demonstrate the effectiveness of RFARN in the vascular segmentation task, quantitative comparisons of accuracy, sensitivity, specificity, and *F*1_*score*_ results are performed on the DRIVE, STARE and CHASE datasets with some state-of-the-art unsupervised and supervised methods. At the same time, the retinal vessel segmentation results of the different unsupervised and supervised methods were visually compared with the retinal vessel segmentation results of the methods in this paper by visualization. Table 5 shows the evaluation metrics for the different retinal vessel segmentation results on the DRIVE dataset. Figs 15 and 16 show the visual comparison of unsupervised and supervised retinal vessel segmentation results on the DRIVE dataset, respectively. Table 6 shows the evaluation metrics for the different retinal vessel segmentation results on the STARE dataset. Figs 17 and 18 show a visual comparison of unsupervised and supervised retinal vessel segmentation results on the STARE dataset, respectively. Table 7 shows the metrics for the evaluation of different retinal vessel segmentation results on the CHASE dataset. Figs 19 and 20 show a visual comparison of unsupervised and supervised retinal vessel segmentation results on the CHASE dataset, respectively. From the experimental results in the graphs, it can be seen that the segmentation performance of the supervised methods is generally better than which of the unsupervised methods. Moreover, the visualization results of the supervised methods are closer to those of expert segmentation. The main reason for this may be the enhanced image intensity or the use of manually designed features to predict the class label of each pixel in the image which provides convenience for the whole segmentation task. Furthermore, learning a set of rules for extracting blood vessels based on the training set makes the network more powerful than unsupervised methods for extracting blood vessel features.

**Table 5:** Comparison of proposed methods with other methods in the DRIVE dataset.

Type	Methods	Year	Acc	Se	Sp	F1
*Unsupervised methods*	2nd human expert		0.9637	0.7743	0.9819	0.7889
	Miao et al. [12]	2015	0.9597	0.7481	0.9748	-
	Azzopardi et al. [15]	2015	0.9442	0.7655	0.9704	-
	Chen et al. [19]	2017	0.9390	0.7358	0.9680	-
	Shah et al. [16]	2019	0.9470	0.7760	0.9724	-
	Tian et al. [17]	2019	0.9580	0.8639	0.9690	-
	Jainish et al. [18]	2020	0.9657	**0.9890**	0.7900	-
*Supervised methods*	Feng et al. [22]	2017	0.9560	0.7811	0.9839	-
	U-Net [21]	2018	0.9531	0.7537	0.9820	0.8142
	Hu et al. [23]	2018	0.9533	0.7772	0.9793	-
	R2U-Net [24]	2018	0.9556	0.7792	0.9813	0.8171
	DUNet [25]	2019	0.9566	0.7963	0.9800	0.8237
	HAnet [26]	2020	0.9581	0.7991	0.9813	0.8293
	Sine-Net [27]	2020	0.9685	0.8260	0.9824	-
	FANet [1]	2021	0.8189	-	0.9826	0.8183
	Huang et al. [3]	2021	0.9701	0.8011	**0.9849**	-
	RFARN (ours)	2021	**0.9712**	0.8788	0.9803	**0.8453**

**Table 6:** Comparison of proposed methods with other methods in the STARE dataset.

Type	Methods	Year	Acc	Se	Sp	F1
*Unsupervised methods*	2nd human expert		0.9522	0.9017	0.9564	0.7417
	Miao et al. [12]	2015	0.9532	0.7298	0.9831	-
	Azzopardi et al. [15]	2015	0.9497	0.7716	0.9701	-
	Chen et al. [19]	2017	0.9390	0.7449	0.9690	-
	Shah et al. [16]	2019	0.9409	0.8004	0.9644	-
	Jainish et al. [18]	2020	0.9657	**0.9900**	0.8500	-
*Supervised methods*	U-Net [21]	2018	0.9690	0.8270	0.9842	0.8373
	Hu et al. [23]	2018	0.9632	0.7543	0.9814	-
	R2U-Net [24]	2018	0.9712	0.8298	0.9862	0.8475
	DUNet [25]	2019	0.9773	0.8369	0.9888	0.8485
	HAnet [26]	2020	0.9673	0.8186	0.9844	0.8379
	Sine-Net [27]	2020	0.9711	0.6776	0.9946	-
	Huang et al. [3]	2021	0.9683	0.6329	**0.9967**	-
	RFARN (ours)	2021	**0.9822**	0.8874	0.9891	**0.8707**

**Table 7:** Comparison of proposed methods with other methods in the CHASE dataset.

Type	Methods	Year	Acc	Se	Sp	F1
*Unsupervised methods*	2nd human expert		0.9733	0.8313	0.9829	0.7969
	Azzopardi et al. [15]	2015	0.9387	0.7585	0.9587	-
	Tian et al. [17]	2019	0.9601	0.8778	0.9680	-
*Supervised methods*	U-Net [21]	2018	0.9578	0.8288	0.9701	0.7783
	Hu et al. [23]	2018	0.9533	0.7772	0.9793	-
	R2U-Net [24]	2018	0.9634	0.7756	0.9820	0.7928
	DUNet [25]	2019	0.9610	0.8155	0.9752	0.7883
	HAnet [26]	2020	0.9670	0.8239	0.9813	**0.8191**
	Sine-Net [27]	2020	0.9676	0.7856	0.9845	-
	FANet [1]	2021	0.7722	-	0.9830	0.8108
	RFARN (ours)	2021	**0.9780**	**0.8352**	**0.9890**	0.8185

To more objectively compare and evaluate the performance of RFARN, we implemented U-Net, R2U-Net, and DUNet networks, which are still very popular models for retinal vessel segmentation tasks. And we compared them by visualization. For the unsupervised method, Miao et al. [12] performed simulation experiments on MATLAB R2012b on a Windows 7 system with 2G memory. Azzopardi et al. performed experiments via Matlab on a personal computer equipped with a 2GHz processor. The hardware environment required for the experiments of this method is less demanding, but its use of two B-COSFIRE converter response summation makes the choice of experimental parameters extremely demanding [15]. Shah et al. completed using an unoptimized Matlab script with an average time of less than 20 seconds per image [16]. Tian et al. used a 1080ti graphics card for training and testing with the software environment PyTorch. The initial learning rate of the method was set to 0.01 and reduced to 90% of the original learning rate after every 20 epochs. After 200 epochs, the network reaches convergence and stops training, and the entire training time of the network is about half an hour [17]. Jainish et al. extracted retinal vessels on MATLAB using probabilistic modeling and expectation of maximum entropy [18]. The method suggested by Chen et al. required only a few experiments in MATLAB 2014 bona 2.4 GHz PC applying CGLI model [19]. In the supervised approach, Hu et al. conducted experiments on hardware configured with two Intel Xeon E5-2650 CPUs and eight NVIDIA GTX1080 GPU graphics cards via the RCF framework of Cae. During the training phase, the learning rate was reduced every 2,000 steps to ensure convergence and the maximum number of iterations was set to 6,000 steps [23]. HANet was implemented on a single Nvidia GeForce Titan X GPU via PyTorch. The method uses Adam’s algorithm for gradient descent with initial learning rate values set to 3*10-4, betas = (0.9, 0.999), and a training batch size of 10 [26]. Sine-Net is implemented on a Xeon(R) CPU E5-2667v4 (3.20 GHz) CPU with 128 GB RAM and NVIDIA Tesla P100 GPU (Graphics Processing Unit) to train the data using TensorFlow’s Keras library. Each training contains 50 epochs of stochastic gradient descent (SGD) followed by 20 epochs of Adam optimizer [27]. FANet all experiments were performed on Volta 100 GPUs and NVIDIA DGX-2 systems using the PyTorch 1.6. framework. The model was trained with 100 epochs using the Adam optimizer (empirically set) with a learning rate of 1e-4 for all experiments, except for the (DRIVE) and CHASE-DB1 datasets, which were tuned to 1e-3 due to the small size of the training dataset [1]. Huang et al. conducted experiments via Pycharm (python 3.6), Keras and its TensorFlow port base with a hardware environment of Intel(R) Core(TM) i7-7700HQ CPU @ 2.81 GHz, 16 GB of RAM, a GPU graphics card of NVIDIA GeForce GTX 1050, and an operating system is 64-bit Windows 10 [3], and the model training is performed using a cross-entropy loss function, optimized by the Adam algorithm. After a specific comparison, we found that the experimental environment requirements of the phase supervised methods are generally higher than those of the unsupervised methods.

### Retinal segmentation results of different methods on the DRIVE dataset

Comparing the vessel segmentation results of different methods, it can be seen from Fig 15 that most of the unsupervised methods have noisy visualization and incomplete vessel segmentation. Among them, the method of Miao et al. [12] using a matched filter with a 2D Gaussian kernel has better segmentation results than the method of Tian et al. [17] using a Gaussian low-pass filter and a Gaussian high-pass filter. By looking at Fig 15, it can be noticed that Tian et al. mis-segmented the optic disc into vessels. In addition, Jainish et al. [18] used an expectation-maximization algorithm with maximum entropy in unsupervised methods to extract retinal vessels. The sensitivity of their segmentation results reached optimal values on both the DRIVE and STARE datasets. The visualization aspect shows that their method has a more accurate extraction of the vessel contours. However, its method segmented vessels with severe breaks and incomplete ends. Compared to the unsupervised vessel segmentation results, RFARN removes most of the noise from the images and provides a more complete segmentation of small vessels.

Comparing the experimental results of different supervised methods, Fig 16 shows that the patch-based fully convolutional neural network of Feng et al. [22] segmented more complete blood vessels with a greater sensitivity compared to U-Net [21]. The same U-shaped architecture of DUNet [25] and Sine-Net [27], the latter of which takes an up-sampling followed by down-sampling approach, this method largely compensates for the shortcomings of partial capillary segmentation in the former method. In addition to this, the HAnet [26] and Huang et al. [3] methods are also improved U-shaped networks, with the difference that HAnet is designed with multiple decoders focusing on features in different regions. Although HAnet is not as accurate as of the segmentation of Huang et al.’s method, the vascular continuity of its method segmentation is stronger than that of the latter. In contrast, the Huang et al. method focuses more on the features of fine vessels, which makes the network more sensitive to all pixel points of the fundus image with an optimal specificity of 0.9849. Compared to other methods, RFARN uses MSRCR preprocessing to focus on vessel features from both a local and global perspective, which improves the network’s ability to extract vessel trunks and ends as well as capillaries from fundus images. RFARN achieved a segmentation accuracy of 0.9712 and an *F*1_*score*_ of 0.8453 on the DRIVE dataset. From a visualization point of view, RFARN fixes some of the method’s vessel segmentation breakdowns and reduces some of the detailed mis-segmentations. It would also be beneficial to avoid these situations as much as possible for the diagnosis of some ophthalmic diseases.

### Retinal segmentation results of different methods on the STARE dataset

On the STARE dataset, it can be found from the visualization results in Fig 17 that Shah et al. [16] proposed a pre-processing method using Gabor wavelets to enhance the green channel of the image, but its metrics for vessel segmentation results on the STARE dataset were not the best when compared with other unsupervised methods. However, observing its visualization results reveals that the method has better segmentation results for vessel ends than the method of Miao et al. [12] Secondly, a comparison of the segmentation results of Ground truth and Miao et al. shows that Shah et al.’s method has some unsegmented trunk vessels, which may be the reason for its poor segmentation accuracy. In addition, Chen et al. [19] improved the problem of low image contrast by mixing the Selective Binary and Gaussian Filtering Regularized Level Set (SBGFRLS) models and the Local Binary fitting (LBF) model. Although it does not require high initial conditions, the segmentation results of this method are far inferior to the vessel segmentation results of the supervised method. The segmentation accuracy of RFARN on the STARE dataset is greatly improved compared to the unsupervised method mentioned in the paper. From a visualization perspective, our segmentation results are closer to Ground truth in both global and local regions, indicating that the RCAM and RSAM modules facilitate RFARN to extract vascular features in both channel dimension and spatial dimension in fundus images.

Supervised methods using U-Net [21] as a baseline network on the STARE dataset are still valid for vascular segmentation tasks, as shown in Fig 18, and R2U-Net [24] is based on U-Net incorporating the idea of recursion and the advantages of residual networks. From the experimental results, R2U-Net provides better verification accuracy than U-Net. Some methods are not based on UNet, such as the method proposed by Hu et al. [23] that applies fully connected conditional random fields (CRFs) based on convolutional neural networks (CNNs) for final segmentation. This method may also affect the final segmentation results if it does not detect poorly the boundaries of vessels with little difference in thinness and thickness. In addition, the experimental results comparing the methods of DUNet and Huang et al. showed that DUNet is more accurate and sensitive than the latter, and its visualization results are more complete. Although the segmentation method of Huang et al. had severe breakage of blood vessels, its ability to extract fine vessels was significantly better than that of DUNet. Its specificity values were optimal on the STARE dataset. By comparison with other methods, RFARN performs better on the STARE dataset in terms of both vessel integrity and continuity. Its segmentation of vessel boundaries and fine vessels is closer to the true value. In terms of specific evaluation metrics, the segmentation accuracy of RFARN was 0.9822 and the *F*1_*score*_ was 0.8707.

### Retinal segmentation results of different methods on the CHASE dataset

By looking at unsupervised vessel segmentation methods in recent years, we find that most methods tend to use a form of matched filtering. For example, on the CHASE dataset, Azzopardi et al. [15] added a selective response operation based on the existing Combination of shifted filter responses(COSFIRE) method, which makes the method somewhat restrictive as its selectivity is determined from the original modalities of the vessel in an automatic configuration process. In contrast, Tian et al. [17] used two filters to obtain low-frequency images and high-frequency images for subsequent feature extraction, respectively, and compared with the method of Azzopardi et al, Tian et al’s method greatly improved the accuracy of vessel segmentation and the sensitivity of the vessels. In addition, by observing Fig 19, it can be found that RFARN and HAnet [26] are obviously segmented more accurately than Tian et al. and the continuity of some capillaries is more complete, and the *F*1_*score*_ of HAnet reaches the highest value of 0.8191. This also indicates that the supervised method is more effective than the unsupervised method for vessel segmentation.

To compare the differences between supervised methods, this paper also presents the experimental results of the CHASE dataset in graphical form. From the visualized results in Fig 20, R2U-Net [24] and DUNet [25] outperform U-Net [21] for the segmentation of fine vessels. However, the higher accuracy achieved by R2U-Net and DUNet also introduces noise and has the effect of segmenting background areas like blood vessels. Such a problem is also seen in Sine-Net [27], where the noise generated in the segmented images is even worse. This does not help the ophthalmologist to diagnose the disease. In contrast to them, RFARN uses good image enhancement techniques to improve the CHASE dataset with uneven background illumination, poor vascular contrast, and stenosis between arteries, which provides a good basis for subsequent segmentation of the model. In terms of visualization, RFARN does not produce as much noise as the other methods of segmentation. And it is cleaner for the ends of fine vessels, and the trunk of the vessel is not affected by too many background factors. In addition, RFARN has improved the accuracy, sensitivity, and specificity of blood vessel segmentation on the CHASE data set, which also benefits from the effective RCAM and RSAM modules. The accuracy and sensitivity of RFARN reached 0.9780 and 0.8352 respectively. Ablation experiments were performed on the DRIVE, STARE and CHASE datasets in comparison with existing retinal vascular methods, and the differences between the results of each method were analyzed in further detail to demonstrate which the method can achieve effective and accurate segmentation of retinal vessels.

### Analysis the number of model parameters and computation time

To illustrate the superiority of this paper’s method in terms of the spatial and temporal spending of the model, we calculated the structure of this paper’s ablation network and the number of parameters of U-Net, R2U-Net, and DUNet in the same experimental setting, and recorded the training time and single image testing time of the network on different datasets. Besides, in order to judge the superiority of vascular segmentation comprehensively, we use a more meaningful measure of the quality of pixelated segmentation, Matthews correlation coefficient(MCC), which considers TP, TF and TP and FN, and is usually considered as a more balanced accuracy indicator. Its calculation is shown in Eq (19):

$$\begin{array}{c} MCC=\frac{TP\times TN-FP\times FN}{\sqrt{\left(TP+FP)\times (TP+FN)\times (TN+FP)\times (TN+FN\right)}} \end{array}$$ (19)

The value of MCC ranges from [-1, 1], where a value of 1 indicates that the prediction is completely consistent with the actual result, a value of 0 indicates that the predicted result is not as good as the random prediction result, and -1 indicates that the predicted result is completely inconsistent with the actual result. Thus, MCC essentially describes the correlation coefficient between the predicted and actual results. Table 8 records details of the number of parameters, time cost and Matthews correlation coefficient (MCC) for different models. In the same experimental setting, U-Net is at a low level of time overhead and number of parameters due to its simple network structure. However, R2U-Net uses a more dense structure for better segmentation effect but makes the number of network parameters relatively high and the time overhead is also higher. Comparing RFARN and DUNet, we can find that RFARN has a higher number of parameters, but its average time of segmentation is slightly faster. By comparing the number of parameters and computation time of each model, we found that the number of parameters varied by the model has an impact on the time of both network training and testing. By further comparing the MCC metrics of different models on the same dataset, we found that the test results of RFARN are better, which also indicates that the vascular segmentation results of RFARN are closer to the true labels. Among them, the MCC values of RFARN reached 0.8302, 0.8480, and 0.7944 on the DRIVE, STARE, and CHASE datasets, respectively. Compared with the ablation experiment ResUNet, it improved by 4.52%, 4.7%, and 5.1%, respectively.

**Table 8:** Details of the number of parameters, time cost and Mathews correlation coefficient (MCC) for different models.

Method	Params	DRIVE			STARE			CHASE		
		Train(s)	Test(s)	MCC	Train(s)	Test(s)	MCC	Train(s)	Test(s)	MCC
*ResUNet*	7.87M	14.20	**3.12**	0.7850	22.42	**10.36**	0.8010	19.97	11.16	0.7434
*ResUNet* + *MSRCR*	8.03M	15.42	4.58	0.7920	23.81	12.43	0.8214	23.27	14.67	0.7889
*ResUNet* + *RCAM*	9.27M	18.44	5.27	0.8229	25.32	12.92	0.8333	24.49	17.90	0.7925
*ResUNet* + *RSAM*	9.49M	20.50	5.42	0.8195	25.99	13.71	0.8363	26.80	17.29	0.7920
*U*−*Net*	7.76M	**14.03**	2.73	0.8094	**19.44**	12.50	0.8165	**11.28**	**9.6**	0.7716
*R*2*U*−*Net*	20.94M	43.20	13.19	0.7852	50.75	49.45	0.8206	35.09	25.60	0.7293
*DUNet*	**4.41M**	20.40	15.21	0.8077	25.48	18.32	0.8251	53.69	47.86	0.7758
*RFARN*	9.74M	23.97	5.75	**0.8302**	27.07	15.54	**0.8480**	30.73	18.71	**0.7944**

### Limitations of current work and directions for future research

Comparing the sensitivity metrics of the DRIVE and STARE datasets, the segmentation results of the CHASE dataset are severely affected by the poor image quality. In addition to this, the negative pixels (non-vascular) of the lesioned fundus image account for a larger proportion of the image, which may be a factor that reduces the network’s ability to extract vascular features. In the future, we can introduce a class balance loss function to improve and accelerate the network training class balance. One more point is that the network structure in the experiments may come at the expense of time and storage in order to achieve more accurate vessel segmentation results, which requires us to further optimize the network model to reduce the reliance on hardware facilities.

In addition, the limited number of existing publicly available fundus image datasets places a significant constraint on the training of the model. In subsequent research work, we can use a suitable number of Ground truth images to supervise the network training to overcome the situation where the model is under-trained due to the small amount of data. Apart from this, good retinal image pre-processing methods remain one of the important directions to explore for future research on medical image segmentation tasks.

## Conclusion

Automatic segmentation of retinal vessel images plays an important role in the diagnosis and screening of disease. Because the complex structure of fundus imaging makes segmentation substantially more difficult, in order to alleviate the problems of low image contrast, illumination limitation and complex connections between blood vessels, this paper firstly enhances the features of blood vessels in fundus images by the multiscale retinex with color restoration (MSRCR) retinal image preprocessing method based on Retinex theory. The pre-processed fundus images are then fed into the Reverse Fusion Attention Residual Network (RFARN) constructed in this paper, and the trained RFARN model is used for testing to achieve automatic segmentation of the final fundus retinal blood vessels. To solve the problem of insufficient accuracy of retinal vessel segmentation, this paper embeds the RCAM module in RFARN to give more attention to the deeper features which are not prominent in the bottom and residual encoders. In addition, the RSAM module is used to enhance the local response of decoder features in the spatial dimension. Retinal vessel segmentation experiments were performed on the DRIVE, STARE and CHASE fundus image databases to evaluate the performance of the *F*1_*score*_, accuracy, sensitivity, specificity, and AUC metrics of RFARN. Compared with some existing retinal vessel segmentation methods such as DUNet, Sine-Net, and HAnet, the RFARN proposed in this paper significantly improves the segmentation of vessels. Given the high accuracy, *F*1_*score*_, and sensitivity of the experimental results, RFARN is effective for retinal vessel segmentation and the method can significantly reduce the misdiagnosis rate of fundus diseases by ophthalmologists.
